# The hidden route: an exploratory study on autonomic influences in early phases of information processing

**DOI:** 10.1186/s40359-025-02561-y

**Published:** 2025-03-13

**Authors:** Elisa Cainelli, Stefano Vicentin, Giulia Stramucci, Sara Guglielmi, Maria Devita, Luca Vedovelli, Patrizia Bisiacchi

**Affiliations:** 1 Department of General Psychology, University of Padova, Via Venezia, 8, 35131 Padova, Italy; 2 Unit of Biostatistics, Epidemiology, and Public Health, Department of Cardiac, Thoracic, Vascular and Public Health Sciences, University of Padova, 35131 Padova, Italy; 3 Padova Neuroscience Center, PNC, 35131 Padova, Italy

**Keywords:** Sensory gating, P50, HRV, Vagus, Autonomous nervous system, Auditory processing

## Abstract

**Background:**

Adapting to an ever-evolving world and the constant changes taking place in one’s own body requires a great deal of regulatory effort in which the brain and periphery act in synergy. In this framework, heart rate variability (HRV) is thought to reflect autonomic regulatory adaptions to the environment. The hypothesis of this exploratory work is that the sensory gating (SG) evoked potential might represent an index of early phases of the cognitive counterpart. This study aimed to investigate the possible association between the two measures in young adults.

**Methods:**

An ECG and a 32-channel EEG were recorded in 32 young adults (mean age 24.1 years, range 20–29) at rest and during an auditory SG paradigm. The peak amplitude for the first (S1) and second (S2) stimulus and the S2/S1 ratio of SG on central site (Cz) were calculated. HRV components in two frequency (low-LF and high-HF) domains and respiration frequency rate (EDR) estimation were calculated from ECG. Smoke habits were collected.

**Results:**

LF HRV component resulted associated with S2/S1 ratio and S2 (S2, rho=-0.498, *p* = 0.02; S2/S1, rho=-0.499, *p* = 0.02), while smoking with S2/S1 ratio (rho=-0.493, *p* = 0.02) and EDR only near significance with S2/S1. In the regression, LF, EDR, and smoke resulted in good predictors of the S2/S1 ratio (LF, Beta=-0.516, *p* < 0.001; EDR, Beta=-0.405, *p* = 0.002, smoke, Beta=-0.453, *p* < 0.001). Applying a machine learning approach showed that the LF HRV component was significantly influenced by frontocentral spectral EEG activity in theta and gamma frequencies.

**Conclusions:**

Even if preliminary, these results suggest a filtering mechanism that operates throughout circuits strongly associated with those generating HRV to adapt to the outside world synergistically.

**Supplementary Information:**

The online version contains supplementary material available at 10.1186/s40359-025-02561-y.

## Background

The role of the autonomous nervous system (ANS) in brain processes has been extensively explored in the last decade (for a review, see [1, 2]). ANS measures, and in particular those associated with parasympathetic activity, have been found to correlate with cognitive, psychological, and behavioral variables, such as attention, executive functions, and emotional control [3–7]. In order to explain the complex interactions between peripheral and central structures and functions, the Neurovisceral Integration Theory has been proposed [7, 8], an integrative effort to combine cognitive processes, autonomic function, and health outcomes into a network of reciprocally connected brain regions called the “central autonomic network” (CAN; [9]).

Heart rate variability (HRV), a measure of the change in the time between heartbeats, is thought to reflect autonomic function, and it is the most used technique in studies interested in autonomic-central interactions. The reason for the popularity of this technique is its non-invasive nature and ability to reflect, although in an non-specific manner, the interdependent inputs between central and autonomic regulatory systems [2, 10]. However, despite the explanatory effort and the growing comprehension of the dynamics involved, several mechanisms remain speculative, and an experimental elaboration about the processes involved needs to be improved.

Neurophysiological techniques such as EEG and evoked response potentials may be useful for exploring these mechanisms because they are direct measures of brain electrophysiology, modulated in real-time by the activity in neural systems. For this reason, neurophysiological techniques are a more accurate method of studying different levels of cognitive functioning than behavioral tests that return a global measure of the subject’s performance.

Among them, the so-called “sensory gating” evoked potential represents a window of the brain at work during an early phase of information processing - the filter of irrelevant information [11–14]. In this paradigm, participants are presented with two identical simple auditory stimuli in the sensory gating task while an EEG response is recorded. The first auditory stimulus (S1) elicits a consistent EEG-measured brain response, which, in adults, occurs approximately 50 msec after the stimulus, the P50 wave. When identical auditory stimuli are presented 500 ms apart, the evoked response to the second stimulus (S2) is generally reduced relative to the response to the first stimulus (i.e., the response is “gated”) [15]. When the response to the second stimulus is not reduced, this is considered a sign of poor sensory gating and impaired cerebral inhibition [14, 16–18], which is crucial for efficient cognitive functioning. As confirmation, many psychiatric illnesses are associated with poor sensory gating (e.g., attention deficit-hyperactivity disorder and schizophrenia [15, 19, 20]). The ability to filter only relevant information, avoiding overloading the system with unnecessary information, is a crucial preliminary process in the complex system of operations adopted to interact and thus adapt to the environment [21, 22].

Considering this preliminary background, we hypothesize that the autonomic nervous system, in its continuous activity of regulating and adapting to internal and external demands, may influence the early information filtering mechanism, which is, in fact, the cognitive component straddling between the external requests and the higher cognitive processes. Specifically, it has been hypothesized that higher parasympathetic activity, which reflects better prefrontal regulatory ability [23, 24], could be associated with improved sensory gating. The sensory paradigm has been studied mainly in clinical populations; the aim of this study is to investigate how sensory gating is associated with HRV measures in healthy rest participants to increase our understanding of the basic mechanisms of autonomic influences on early phases of information processing. Other factors could influence the results, in particular respiration and smoking. Therefore, that data has been collected and added to the analysis: respiration can contribute to the generation of HRV [25, 26], so we also evaluated the role of an estimation of respiration rate (EDR) on SG; smoking can influence the sensory gating [25, 26]; therefore, its effect has also been considered.

Finally, to seek further confirmation about the association between autonomic and central activity, it has been explored how EEG activity at rest could influence LF and HF HRV parameters. Starting from the Neurovisceral Integration Theory, it has been hypothesized that EEG activity, particularly that of frontal sites, could be associated with HRV, suggesting a prefrontal involvement in autonomic control.

## Methods

### Participants

Thirty-four healthy undergraduates from the University of Padua volunteered for the study. Three participants were excluded due to artifacts in the sensory gating ERP; therefore, the final sample consisted of 31 participants (14 males, 17 females; mean 24.3 years, range 20–28). The sample size was defined through a power analysis (effect size f = 0.25, power b=0.95) conducted with the software G*Power [27] and based on previous studies with healthy participants [28]. Cardiovascular or neurological diseases, as well as drug intake, were exclusion criteria. Participants were asked to avoid eating at least two hours before the recording, drinking coffee or alcohol, smoking, or doing strenuous physical exercise. Smoking habits have been explored with a modified version of the Italian ministerial “no smoking” questionnaire [29]. The University of Padua Ethics Committee approved the study (Protocol 103-c, approved on 20th October 2023), and all volunteers expressed written informed consent to participate. The study was conducted following the Declaration of Helsinki. The population characteristics are shown in Table 1.

**Table 1:** Characteristics of the populations

Population characteristics	
Age, median (Q1; Q3)	24 (23; 25.5)
Sex, M:F	14:17
Smokers, N/tot (%)	9/31 (29%)
Faculty, N/tot (%)	
Psychology	15/31 (48%)
Medicine	4/31 (13%)
Nursing	3/31 (10%)
Other	9/31 (29%)

### Procedure

Upon arrival at the laboratory, participants received general information about the experiment and read and signed the informed consent. Then, each participant was seated in a comfortable armchair in a sound-attenuated, dimly lit room. Before the beginning of the experimental procedure, participants underwent a preparatory procedure, which consisted of measuring the scalp circumference (to select the EEG cap that best fit each individual’s head shape) and collecting demographic information (age, gender, educational level). Subsequently, forehead and mastoid sites were cleaned to remove skin and makeup residue. Similarly, two sites were detected and cleaned to apply the ECG electrodes, corresponding to the area beneath the right clavicle and below the left ribcage. The selected elastic EEG cap (32 channels) was positioned on the participant’s head and connected to the EEG amplifier. After the application procedure, each participant completed a 5-minute resting-state recording. Participants were instructed to reduce their movements and to focus on a white fixation cross displayed at the center of a grey screen to reduce muscular and ocular artifacts. After this first block, participants underwent the auditory sensory gating paradigm.

### Electrophysiological data recording and processing

#### ECG data collection and processing

Ag/AgCl surface electrodes were positioned on the chest in a modified lead II configuration to register the ECG. The raw ECG signal was recorded with a LiveAmp system (Brain Products, Gilching, Germany) and amplified with a gain of 150, bandpass filtered (0.3–100 Hz), and digitized at 500 Hz (16 bit A/D converter; resolution 0.559 µV/LSB). The raw ECG signal was then exported to Kubios HRV Analysis Software 2.2 (The Biomedical Signal Analysis Group, Department of Applied Physics, University of Kuopio, Finland) to estimate the occurrence of each heartbeat and derive the series of inter-beat intervals (IBIs), calculated as the difference in milliseconds (ms) between two consecutive R-waves. Fast Fourier spectral analysis was conducted on the IBI series to compute frequency domain indexes, in particular HF power (0.15–0.40 Hz), which reflects parasympathetic activity and is labeled “the respiratory band” because it corresponds to the HR variations related to the respiratory cycle (RSA), and LF power (0.04–0.15 Hz), associated with parasympathetic and sympathetic activity and with blood pressure regulation via baroreceptors (during resting conditions, LF power is known to reflect mainly baroreceptor activity, see [1]. Frequency-domain indexes were logarithmically transformed to normalize their distribution. An estimate of the respiration frequency is also computed (i.e., electrocardiogram-derived respiration - EDR).

### EEG and sensory gating

The experiment was characterized by 5 min of EEG recording at rest with open eyes, followed by the sensory gating paradigm. Therefore, the sensory gate components were extracted from a multichannel EEG recording. The EEG signal was collected using an elastic cap with tin electrodes (Electro-cap International, Inc.) from 32 scalp positions referenced to the bilateral mastoids. Vertical and horizontal electrooculograms (EOGs) were recorded using a bipolar montage to monitor eye movements and eye blinks. The raw EEG signal was recorded with a LiveAmp system (Brain Products, Gilching, Germany) using the Brain Vision Recorded software. Electrode impedances were kept below 5 kΩ. The digital EEG signal was sampled at 512 Hz and filtered in the [0.5–40 Hz] band. Data was preprocessed using Brainstorm [30]. First, data were visually inspected to remove epochs presenting consistent electrical artifacts. Then, independent component analysis was conducted (method: PICARD) to remove biological and electrical artifacts, such as blinks, muscular movements, and transient distortions.

Auditory stimuli were delivered through headphones over ears (Sony WH-1000XM4) placed on the participants’ heads before the beginning of data acquisition. The SG paradigm was created using the Opensesame software [31] and consisted of the presentation of a couple of clicks at an interval of 500 ms. The average interval between click pairs was 9 s. To reduce predictability and expectations, the length of these windows was pseudorandomized between 8, 9, and 10 s, as in [32]. Each click was presented at an intensity of 75 decibels and was associated with a trigger appearing in the EEG recording with a consistent delay of 2 ms (adjusted).

Triggers in the EEG recording (labeled S1 and S2) were used to define the epochs of interest. Specifically, epochs from 100 ms before to 400 ms after each stimulus were extracted from the recording, applying a baseline normalization focused on the [-200 -2] time window (this interval was chosen to avoid confounding effects of activity associated with S1 on the S2 time window). Subsequently, epochs were carefully inspected and subject to artifact rejection. Specifically, every epoch in which any electrode’s signal exceeded +/− 75 [mu]V was excluded from further analysis. From the initial 100 epochs, an average of 89 ± 5 valid epochs were retained for each condition (S1 and S2).

The procedure to detect the sensory gating effect focused on the electrode Cz because many studies have demonstrated that the most recognizable difference between clicks occurs over this site [33]. For each participant and condition (click1 or click2), activity was averaged between all the good epochs. The resulting time window was visually inspected to identify a definite peak P50 component separately for S1 and S2, following the parameters suggested by Kisley and colleagues [16]. Namely, P50 was defined as the largest point in the measurement window between 40 and 80 ms, surrounded on both sides by lower voltages, as defined by [34]. The amplitude of P50 was measured using Matlab and corresponded to the difference between the positive peak identified in this interval and the preceding negative trough, consistent with the literature on the topic [16]. Component P50 for the S2 response was defined following the same parameters, with the additional constraint that the positive peak was required to occur within 10 ms before or after the S1 peak. If no peak was detected for S2 within this window, the component was considered completely attenuated, and its amplitude was set to zero, consistent with the literature on the topic [16, 35]. Additionally, the component was required to have a similar latency, with its positive peak occurring within +/− 10 ms from the S1 P50 latency. If no peak existed within this window, the S2 P50 amplitude was set to zero, and the S2 P50 latency was set to the corresponding S1 P50 latency.

SG was measured by comparing the amplitude of the wave evoked by the second click (S2) to the amplitude evoked by the first click (S1). Specifically, a ratio of magnitudes, the S2/S1 ratio, was computed to quantify sensory gating. S2/S1 ratios closer to 0 indicate robust suppression (very small S2 response compared to S1 response), whereas greater ratios indicate essentially no sensory gating (S2 and S1 responses were comparable in magnitude). In healthy adults, S2/S1 ratios for the P50 component range from 0 to over 1 [16, 36].

### Statistical analysis

The correlation between HRV, EDR, and SG measures was calculated using Spearman’s rho correlation. Confidence intervals were calculated using 1000 bootstrap resamples. Multiple comparisons p-values were corrected for false discovery rate. The significant variables at the correlation (LF and smoke; EDR was slightly significant and still considered) were inserted in linear regression as predictors of the S2/S1 ratio.

Finally, to further explore the association between autonomic (HRV) and central activity (EEG) we implemented a feature selection using a machine-learning algorithm based on a random forest (Boruta) [37]. The Boruta algorithm aims to identify all the relevant predictors that impact the outcome of interest (in our case, LF and HF). To add robustness to the feature selection analysis, the Boruta algorithm was iterated through five different initial seeds of the random number generator, and features identified as important in all five iterations were finally kept as important. The dataset was complete.

Statistical significance was set at *p* < 0.05 except for Boruta and correlations, which were set at *p* < 0.01. Statistics were determined using SPSS (I.B.M. SPSS version 29.01) and R (v.4.4.2).

## Results

**Fig. 1:**
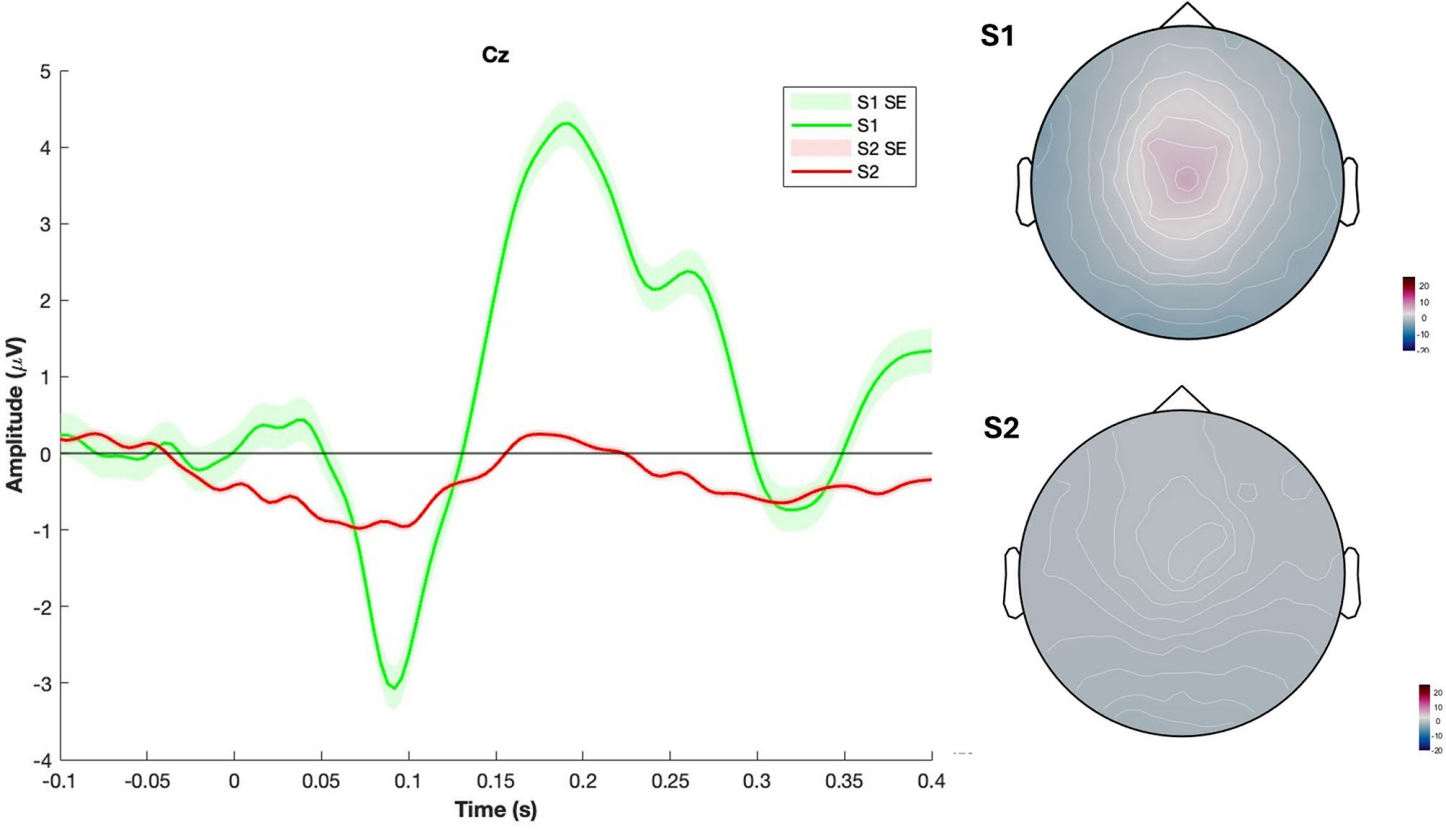
The figure shows the grand mean waveform of the potential evoked by the sensory gating paradigm

Figure 1 shows the grand mean waveform and the topoplot of the potential evoked by the sensory gating paradigm.

No evidence for sex differences in SG, HRV, and EDR measures was found.

The results obtained by the logarithmic transformation of LF and HF are reported in Table 2. Descriptive values of the other HRV parameters are provided in Supplementary Material 1.

**Table 2:** Median and quartile range of the logarithmic LF and HF results

	*N*	Median (Q1, Q3)
HF log	31	6.01 (5.52, 6.80)
LF log	31	7.47 (7.02, 8.05)

Legend: HF: high-frequency; LF: low-frequency

Spearman’s rho correlation was performed between HRV measures, EDR, smoke, and SG components (Table 3).

**Table 3:** Spearman’s Rho correlation between HRV measures, EDR, smoke, and SG components after false discovery rate correction

	S1 rho (95%C.I)	S2 rho (95%C.I)	S2/S1 ratio rho (95%C.I)
LF	− 0.305(-0.612; 0.083, *p* = 0.228)	**− 0.498(-0.757; − 0.149**, *p* = **0.020)**	**− 0.499(-0.768; − 0.153**, *p* = **0.020)**
HF	− 0.290(-0.587; 0.086, *p* = 0.228)	− 0.218(-0.532; 0.173, *p* = 0.408)	− 0.157(-0.473; 0.285, *p* = 0.479)
EDR	− 0.059(-0.351; 0.420, *p* = 0.753)	− 0.137(-0.506; 0.244, *p* = 0.505)	− 0.362 (-0.626; 0.015, *p* = 0.135)
Smoke	0.175(-0.183; 0.501, *p* = 0.463)	− 0.191(-0.502; 0.145, *p* = 0.456)	**− 0.493 (-0.711; − 0.191**, *p* = **0.020)**

Legend: LF: low-frequency; HF: high-frequency; EDR: electrocardiogram-derived respiration. C.I.: confidence interval

**Fig. 2:**
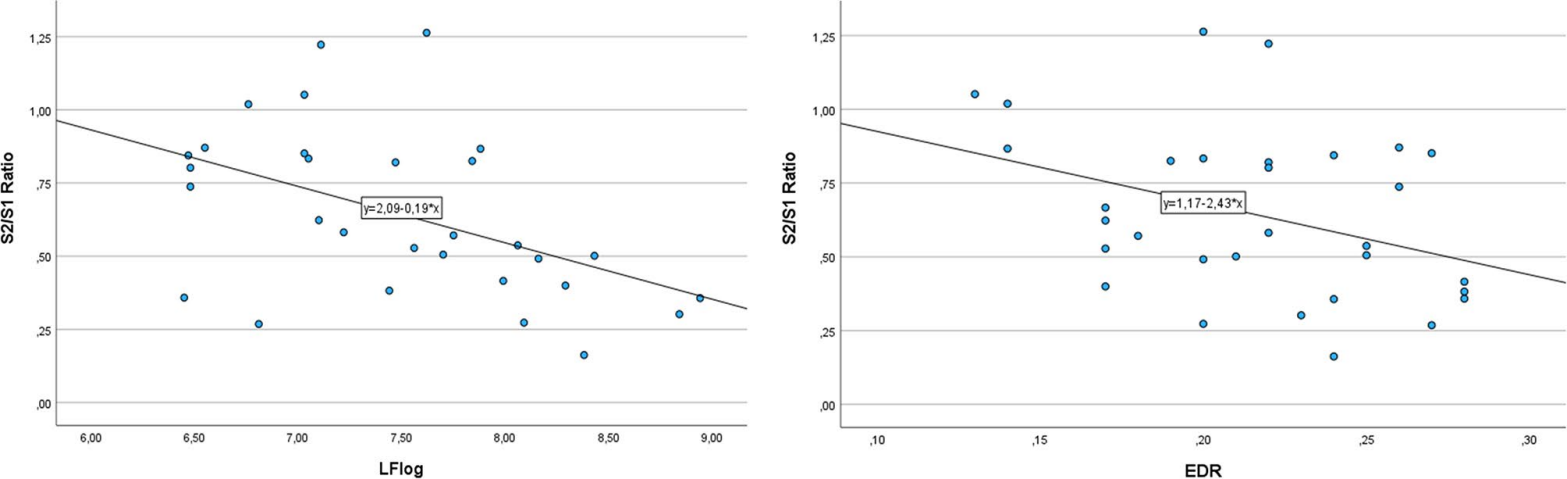
The figure shows the dispersion scatterplots of significant correlations among LF, EDR and S2/S1 ratio

Figure 2 shows the dispersion scatterplots of significant results in LF and EDR.

The variables we found significant in the correlation analysis (i.e., LF and smoke. EDR was near significance and clinically important and thus considered too) were put into linear regression and resulted in good predictors of the S2/S1 ratio (LF, Beta=-0.516, *p* < 0.001; EDR, Beta=-0.405, *p* = 0.002, smoke, Beta=-0.453, *p* < 0.001).

**Fig. 3:**
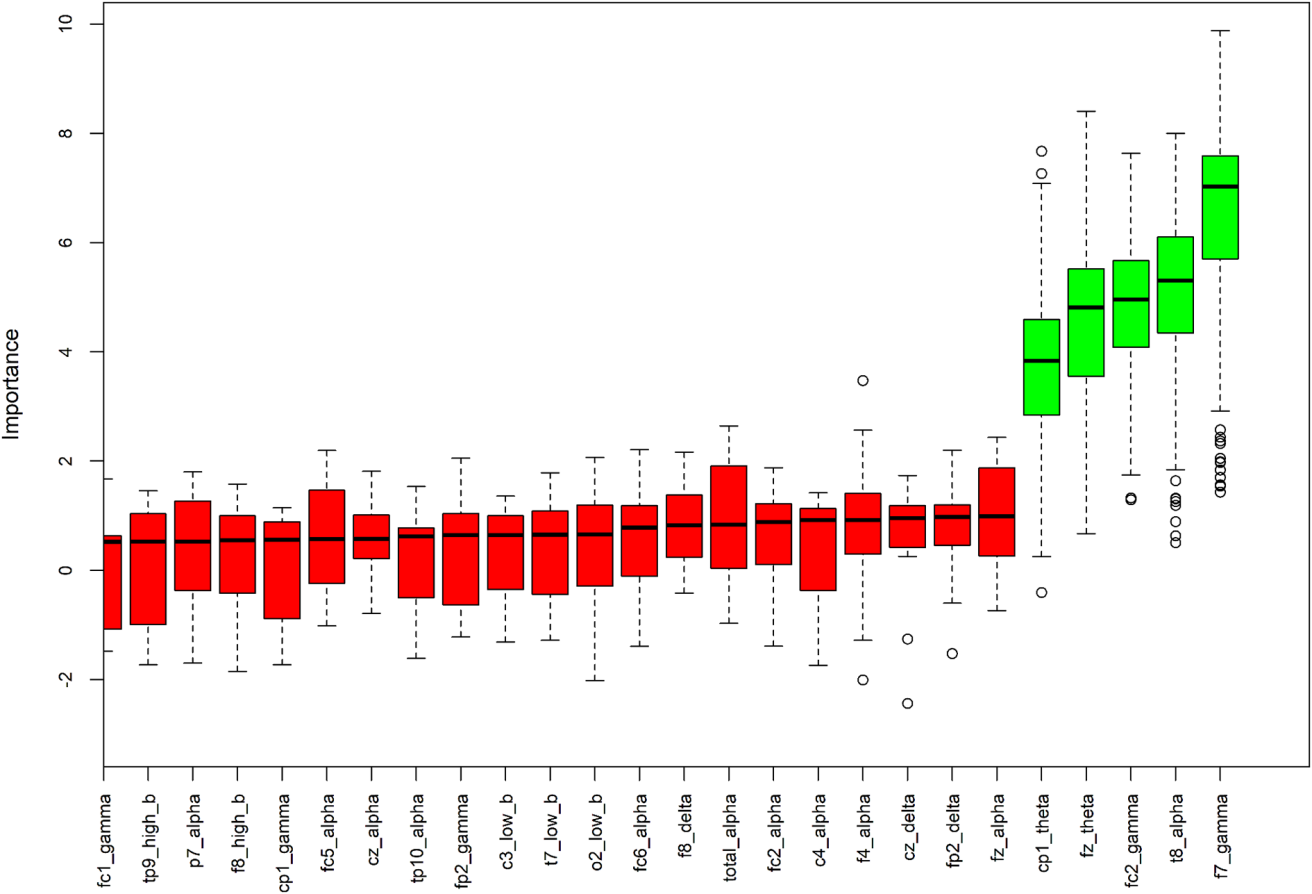
We applied the machine-learning algorithm (Boruta) to find the most influential EEG features accounting for changes in LF results. The algorithm gives an overall index of the importance of each variable with their respective standard errors and a dichotomic evaluation of “important “(green boxes) or “not important” (red boxes). The solid black line represents the mean, the box edges are the first and third quartiles, and the circles are outliers, defined as outside 1.5 times the interquartile range (whiskers) above the upper quartile and below the lower quartile

To further explore the association between autonomic and central nervous systems and find confirmations about the data on sensory gating, we used a machine-learning algorithm (Boruta) to find the most influential EEG spectral power features in determining changes in the LF and HF HRV components of participants. The algorithm gave an overall index of the importance of each variable with its respective standard errors and a dichotomic evaluation of “important “ or “not important.“ We preliminarily put into the algorithm the data coming from all the electrode sites for all the frequency bands, obtaining a clear predominance of important variables in frontal sites in the gamma and theta bands (Fig. 3). Complete results on iterations are available in the Supplementary Material 2. The features identified as important in all five iterations for LF were CP1 and Fz in theta, F7 and FC2 in gamma, and T8 in alpha (see Fig. 3). There are no important components repeating for all five iterations for HF (Fig. 3).

## Discussion

This work wanted to explore how early phases of information filtering (sensory gating) and peripherical measures, such as HRV and respiration, interact in healthy young adults. EDR (respiration) and smoke have been found to be inversely correlated with the S2/S1 ratio and LF both with the S2 component and the S2/S1 ratio. The linear regression shows that they are also good predictors of the sensory gating process: at higher LF values and respiration rate, and in smokers, a lower S2/S1 ratio (better filtering) was associated. Finally, as a confirmation of the contribution of HRV in the brain processes, we explored with a machine learning approach the association between background EEG and HRV parameters, finding it on LF HRV component and fronto-central EEG sites.

In our study, both the sensory gating paradigm and the analysis of at-rest EEG highlighted the LF HRV component as the only one involved. In contrast, nothing emerges from the HF parameter, which is usually the most studied component in psychological research because it is thought to reflect vagal-cardiac nerve activity and demonstrated associations with a broad range of cognitive tasks [5, 38–41]. Literature often reports also results in the LF component of HRV, but this frequency band obtained less attention because of its indefinite origin and role and variability according to the experimental condition [1, 2]. In short-term recordings, upright sitting positions, and resting conditions (as in the present study), LF is thought to reflect parasympathetic and baroreflex activity [42, 43]. Given its dissociated effects from the component that, by definition, represents the respiratory sinus arrhythmia and thus parasympathetic activity, namely HF, it probably sums up multiple forces at work, as in general, many other HRV measures do. Therefore, in our experimental condition, the lower frequencies of the HRV spectrum caught up with some variation better than HF, both in the at-rest EEG condition and sensory gating paradigm. It was beyond the scope of this work to elucidate the origin and the role of the different HRV bands in brain activity. It is, after all, impossible to identify precise correspondences between cognitive constructs and psychophysiological measures, which only can reflect how the system works. The aim of the present study was rather to investigate the possible role of peripheric influences on the early phases of elaboration processing of stimuli coming from the external world. It has been considered sensory gating particularly suitable for this scope because it indexes an automatic ability to inhibit irrelevant information, avoiding voluntary processes’ confounding influences.

In the present data, higher LF was specifically correlated with reduced S2 and S2/S1 ratio, indicating an association of these HRV oscillations with the filtering process. This result, although it does not exactly clarify the roles of the different actors on the scene, nevertheless testifies to the intricate relationship between heart, breath, and brain, that is, between the autonomic and central nervous systems. The type of analysis performed in our study does not allow us to infer the direction of the relationship nor to rule out bidirectionality between the two systems, but it does suggest that even in early information processing mechanisms, the autonomic system is involved, both in its cardiac and respiratory components.

The present data are preliminary and exploratory, but previous research, although represented by few and scarcely comparable studies, would seem to support our conclusions. It has been shown that stress-induced sympathetic activation [42–46] and modulation of the parasympathetic component affect sensory gating [44, 45]. Interesting insight also comes from clinical research: works on schizophrenia (in which sensory gating is well studied) showed facilitation of sensory gating by increasing parasympathetic activation [46]. Similarly, non-invasive vagal nerve stimulation improves sensory gating [45].

Finally, the data on at-rest EEG seems to strengthen the results on the sensory gating. In fact, it has been found that, specifically for LF, the EEG activity in the theta and gamma bands, mainly on frontocentral sites, accounts for variations in the HRV parameters. This is in line with the Neurovisceral Integration Theory [7, 8] and supports the hypothesis of a network of reciprocally connected brain regions (the CAN; [9]), where the prefrontal sites through the vagus modulate the autonomic-central interplay.

Again, LF emerged as the most involved component, confirming the results on the sensory gating but opening interesting questions about how there are no results on HF and the possibility that the understanding of what catches up by HRV is already nascent. The way ANS interacts with cognitive processes needs to be clarified. The highly interconnected CAN centers, their vagal inputs, and the shared projections to other brain centers create a crucial infrastructure well suited to supporting efficient exchange with the external world. The vagus nerve projects to the central nervous system, where the continuous flow of signals from outside is efficiently processed, offering information about the changing sensory state of the body and the highly variable environment. A crucial step in this process is signal filtering and compression, which increase signal quality and decrease processing cost [21, 47–49], and that is exactly what is caught up by the sensory gating. Therefore, in this context, the present results might indicate that body’s state of regulation and activation, as drived by the heart and breath (i.e., autonomic nervous system), modulates our ongoing brain activity (the EEG) and thus the ability to process information. This would make sense in terms of adaptivity and economy: depending on circumstance, behavioral states (such as sleep, resting wakefulness, and focused attention), information coming from the outside world might have more or less priority.

In this context, respiration is also crucial, given the strong interconnections between cardiac and respiratory networks [50–52]. A confirmation, in the present study, respiration is shown to predict the sensory gating capability. HRV measures arise from a close integration of respiratory and cardiac activity, and one of the most debated issues in recent years was the role of respiration in HRV interpretation [50, 51, 53]. There is broad evidence of systematic changes in respiration across a wide range of psychological states [50, 53, 54] and of an effect of respiration on brain oscillations [55–59]. Furthermore, changes in respiration rate could affect HRV, inducing a possible confounding factor in the interpretation, rendering the recording of this parameter very important. Despite that, respiration is poorly considered by researchers working with HRV [50]. Unfortunately, this is also a limitation of our study. There are better choices than ours to measure respiration: extracting the information from the ECG only allows an estimation (not a measure) of one of the respiration parameters, the rate [60]. Although this simple estimate has shown a good predictivity power on sensory gating, it is unfortunate that we do not have a more reliable and comprehensive measure than simple frequency to understand its influence better.

Finally, nicotine has been shown to improve sensory gating by stimulating nicotinic cholinergic receptors [26]. Cholinergic input stimulates GABAergic interneurons tonically, resulting in enhanced interneuron firing, thus improving sensory gating [61]. Even if it has been shown that nicotine’s modulatory effect should be concluded with our experimental modalities (no smoking for the previous two hours) [62], we found a better sensory gating in smokers. This result suggests possible long-lasting effects in chronic smokers and is in accordance with previous data showing that in the healthy young adult population, P50 sensory gating was significantly better for smokers than never-smokers, with no difference between abstaining and smoking conditions [63].

The present study has several limitations. First of all, the small sample size can only limit the study to a pilot exploration of the topic. Furthermore, the small sample size exposes us to an increased risk of a Type II error even if the power analysis demonstrated an adequate sample size.

The correlational design limits the possibility of examining the direction of the autonomic-central association and discriminating between bottom-up and top-down influences. A concluding remark should be made regarding the choice of study participants. Both HRV and SG are strongly influenced by variables such as age. Therefore, for this study, whose purpose was to identify a basic pattern of functioning, we chose a population of young, healthy adults. It is important to keep in mind that the results are not necessarily generalizable to other populations.

## Conclusions

An association between HRV parameters and both EEG and sensory gating has been found, suggesting a filtering mechanism that operates throughout circuits overlapping with those generating HRV to modulate brain and behavior to the outside world adaptively. We also found an additional modulating effect of other factors (respiration and smoking), suggesting the presence of many forces at play.

The present study, even if exploratory, supports the idea that dynamic neurophysiological regulatory processes, dominated by the ANS, promote ongoing regulation and adaptation in response to environmental and biological challenges or demanding stimuli. The study of the relationship between cognitive mechanisms and ANS responses can help us understand this complex central and autonomic interplay. These findings, although preliminary, offer novel and preliminary insights into the neurophysiological correlations underlying our ongoing adaptive regulatory mechanisms.

## Electronic supplementary material

Below is the link to the electronic supplementary material.

Supplementary Material 1

Supplementary Material 2

Supplementary Material 3

## Data Availability

All data analyzed during this study are included in this published article as supplementary material.

## Abbreviations

ANS: Autonomous nervous system
CNS: Central nervous system
ECG: Electrocardiogram
EDR: Electrocardiogram-derived respiration
EEG: Electroencephalogram
HF: High frequency
HRV: Heart rate variability
LF: Low frequency
SG: Sensory gating
