# Supplementary material for: The hidden route: an exploratory study on autonomic influences in early phases of information processing
Source: BMC Psychol. 2025 Mar 13;13:241. doi: 10.1186/s40359-025-02561-y (PMC11905487; doi:10.1186/s40359-025-02561-y)
Supplement: Supplementary file 2 — Supplementary Material 2 [file 40359_2025_2561_MOESM2_ESM.docx]

| *Descriptive Statistics* | | | | | | | | | |
| --- | --- | --- | --- | --- | --- | --- | --- | --- | --- |
|  | | SDNN | | RMMSD | | LF_HF_Ratio | | Entropy | |
| Valid |  | 31 |  | 31 |  | 31 |  | 31 |  |
| Missing |  | 0 |  | 0 |  | 0 |  | 0 |  |
| Median |  | 64.900 |  | 35.900 |  | 4.320 |  | 1.240 |  |
| Mean |  | 67.066 |  | 44.457 |  | 5.345 |  | 1.238 |  |
| Std. Deviation |  | 25.260 |  | 30.374 |  | 4.800 |  | 0.371 |  |
| Minimum |  | 30.400 |  | 11.300 |  | 0.230 |  | 0.560 |  |
| Maximum |  | 135.500 |  | 141.500 |  | 19.510 |  | 2.240 |  |
| 25th percentile |  | 47.450 |  | 28.750 |  | 2.275 |  | 0.995 |  |
| 50th percentile |  | 64.900 |  | 35.900 |  | 4.320 |  | 1.240 |  |
| 75th percentile |  | 87.400 |  | 46.950 |  | 6.065 |  | 1.480 |  |
|  | | | | | | | | | |
