# Supplementary material for: The hidden route: an exploratory study on autonomic influences in early phases of information processing
Source: BMC Psychol. 2025 Mar 13;13:241. doi: 10.1186/s40359-025-02561-y (PMC11905487; doi:10.1186/s40359-025-02561-y)
Supplement: Supplementary file 3 — Supplementary Material 3 [file 40359_2025_2561_MOESM3_ESM.docx]

# BORUTA

# Data Import e descrittive

Data summary

| Name | sens |
| --- | --- |
| Number of rows | 31 |
| Number of columns | 215 |
| _______________________ |  |
| Column type frequency: |  |
| numeric | 215 |
| ________________________ |  |
| Group variables | None |

**Variable type: numeric**

| skim_variable | n_missing | complete_rate | mean | sd | p0 | p25 | p50 | p75 | p100 | hist |
| --- | --- | --- | --- | --- | --- | --- | --- | --- | --- | --- |
| subject | 1 | 0.97 | 117.74 | 10.68 | 101.00 | 108.50 | 117.00 | 126.50 | 136.00 | ▇▆▆▆▅ |
| sdnn | 2 | 0.94 | 66.98 | 26.90 | 30.35 | 43.40 | 64.03 | 87.76 | 135.55 | ▇▅▅▃▁ |
| rmssd | 2 | 0.94 | 43.59 | 30.93 | 11.26 | 25.17 | 35.94 | 46.54 | 141.49 | ▇▅▂▁▁ |
| vlf | 2 | 0.94 | 1841.07 | 1991.14 | 207.58 | 484.09 | 1006.86 | 2517.47 | 8623.12 | ▇▃▁▁▁ |
| lf | 2 | 0.94 | 2215.08 | 1420.86 | 336.67 | 1140.80 | 2051.18 | 2863.49 | 5414.55 | ▇▇▆▂▂ |
| hf | 2 | 0.94 | 895.13 | 1207.61 | 54.29 | 273.21 | 505.40 | 856.54 | 5558.41 | ▇▁▁▁▁ |
| lf_hf_ratio | 2 | 0.94 | 4.65 | 3.64 | 0.52 | 2.19 | 3.70 | 6.02 | 17.81 | ▇▆▁▁▁ |
| fp1_delta | 0 | 1.00 | 43.44 | 11.49 | 21.69 | 37.87 | 41.58 | 50.03 | 65.15 | ▅▇▇▅▇ |
| fp1_theta | 0 | 1.00 | 18.86 | 7.26 | 1.63 | 15.74 | 20.00 | 22.56 | 32.69 | ▂▁▇▇▂ |
| fp1_alpha | 0 | 1.00 | 17.89 | 12.69 | 0.00 | 11.36 | 14.44 | 20.15 | 50.68 | ▂▇▁▁▂ |
| fp1_low_b | 0 | 1.00 | 7.71 | 3.34 | 0.00 | 6.51 | 7.58 | 9.69 | 15.39 | ▂▂▇▃▁ |
| fp1_high_b | 0 | 1.00 | 6.97 | 5.76 | 0.50 | 3.81 | 5.87 | 7.72 | 28.54 | ▇▅▁▁▁ |
| fp1_gamma | 0 | 1.00 | 5.12 | 4.27 | 0.35 | 2.63 | 4.20 | 6.80 | 21.63 | ▇▃▁▁▁ |
| fp2_delta | 0 | 1.00 | 39.14 | 15.12 | 7.07 | 28.53 | 41.38 | 49.07 | 65.91 | ▂▅▇▆▃ |
| fp2_theta | 0 | 1.00 | 19.89 | 9.56 | 1.45 | 15.41 | 21.31 | 23.05 | 42.82 | ▂▅▇▂▁ |
| fp2_alpha | 0 | 1.00 | 20.53 | 11.39 | 8.42 | 12.75 | 17.42 | 19.64 | 51.43 | ▇▅▁▁▂ |
| fp2_low_b | 0 | 1.00 | 8.38 | 2.90 | 1.04 | 6.47 | 7.75 | 10.36 | 14.48 | ▁▅▇▆▂ |
| fp2_high_b | 0 | 1.00 | 7.18 | 4.80 | 0.42 | 3.98 | 6.33 | 8.86 | 20.99 | ▇▇▃▁▁ |
| fp2_gamma | 0 | 1.00 | 4.88 | 3.15 | 0.31 | 2.60 | 3.92 | 6.52 | 12.21 | ▇▇▆▂▂ |
| f7_delta | 0 | 1.00 | 43.31 | 13.98 | 0.01 | 39.00 | 46.74 | 49.88 | 64.21 | ▁▁▂▇▃ |
| f7_theta | 0 | 1.00 | 20.29 | 7.07 | 2.87 | 17.00 | 21.12 | 23.62 | 43.40 | ▁▃▇▁▁ |
| f7_alpha | 0 | 1.00 | 18.86 | 12.89 | 1.49 | 11.54 | 15.90 | 21.73 | 57.60 | ▆▇▂▁▁ |
| f7_low_b | 0 | 1.00 | 7.39 | 2.47 | 0.83 | 6.47 | 7.68 | 8.71 | 12.75 | ▁▂▇▆▂ |
| f7_high_b | 0 | 1.00 | 6.74 | 3.83 | 3.27 | 4.51 | 5.77 | 7.38 | 21.13 | ▇▂▁▁▁ |
| f7_gamma | 0 | 1.00 | 3.41 | 2.71 | 0.19 | 0.95 | 3.53 | 4.87 | 12.49 | ▇▇▂▁▁ |
| f3_delta | 0 | 1.00 | 37.51 | 15.06 | 2.73 | 31.71 | 39.04 | 48.52 | 58.54 | ▃▁▅▇▆ |
| f3_theta | 0 | 1.00 | 20.99 | 8.34 | 0.00 | 18.13 | 20.68 | 25.04 | 38.61 | ▂▁▇▅▃ |
| f3_alpha | 0 | 1.00 | 19.31 | 11.99 | 1.44 | 13.09 | 16.48 | 20.44 | 60.00 | ▃▇▁▁▁ |
| f3_low_b | 0 | 1.00 | 8.89 | 3.49 | 0.98 | 7.02 | 8.07 | 11.49 | 15.21 | ▁▂▇▃▃ |
| f3_high_b | 0 | 1.00 | 8.26 | 4.87 | 0.33 | 5.49 | 7.12 | 11.36 | 23.20 | ▃▇▃▂▁ |
| f3_gamma | 0 | 1.00 | 5.04 | 4.00 | 0.00 | 2.57 | 4.41 | 6.62 | 19.50 | ▇▇▅▁▁ |
| fz_delta | 0 | 1.00 | 40.65 | 12.96 | 6.88 | 34.74 | 42.22 | 47.70 | 63.57 | ▁▂▅▇▃ |
| fz_theta | 0 | 1.00 | 20.58 | 9.51 | 2.70 | 15.64 | 21.72 | 27.03 | 38.47 | ▃▃▇▆▂ |
| fz_alpha | 0 | 1.00 | 20.07 | 12.74 | 1.37 | 13.45 | 16.20 | 20.92 | 48.47 | ▂▇▂▁▂ |
| fz_low_b | 0 | 1.00 | 8.33 | 3.12 | 0.89 | 6.38 | 7.94 | 9.53 | 14.92 | ▁▅▇▂▃ |
| fz_high_b | 0 | 1.00 | 6.45 | 3.65 | 0.70 | 3.76 | 6.04 | 7.57 | 17.29 | ▆▇▅▃▁ |
| fz_gamma | 0 | 1.00 | 3.91 | 2.98 | 0.26 | 1.63 | 3.70 | 5.38 | 11.04 | ▇▅▅▁▁ |
| f4_delta | 0 | 1.00 | 32.91 | 18.03 | 0.01 | 22.82 | 35.97 | 44.96 | 67.35 | ▃▂▅▇▂ |
| f4_theta | 0 | 1.00 | 24.27 | 10.34 | 2.67 | 18.53 | 21.65 | 31.03 | 59.85 | ▁▇▃▁▁ |
| f4_alpha | 0 | 1.00 | 17.45 | 12.46 | 1.05 | 10.06 | 16.47 | 23.60 | 47.60 | ▆▇▆▁▂ |
| f4_low_b | 0 | 1.00 | 10.02 | 5.47 | 0.00 | 7.47 | 9.21 | 12.59 | 28.37 | ▂▇▅▁▁ |
| f4_high_b | 0 | 1.00 | 8.43 | 5.38 | 0.00 | 4.89 | 8.17 | 11.09 | 27.83 | ▆▇▃▁▁ |
| f4_gamma | 0 | 1.00 | 6.92 | 6.23 | 0.27 | 2.77 | 5.45 | 10.49 | 31.50 | ▇▆▁▁▁ |
| f8_delta | 0 | 1.00 | 43.68 | 16.02 | 2.98 | 38.21 | 46.54 | 55.40 | 68.16 | ▂▁▃▇▆ |
| f8_theta | 0 | 1.00 | 20.59 | 9.04 | 2.76 | 16.70 | 21.29 | 23.41 | 54.68 | ▁▇▂▁▁ |
| f8_alpha | 0 | 1.00 | 18.97 | 12.25 | 1.52 | 11.72 | 15.26 | 22.66 | 55.87 | ▅▇▂▁▁ |
| f8_low_b | 0 | 1.00 | 6.80 | 3.58 | 0.70 | 5.05 | 6.88 | 8.48 | 15.62 | ▃▇▇▃▂ |
| f8_high_b | 0 | 1.00 | 6.14 | 3.43 | 0.33 | 4.23 | 5.61 | 7.53 | 14.84 | ▃▇▅▁▂ |
| f8_gamma | 0 | 1.00 | 3.83 | 2.85 | 0.00 | 2.02 | 3.80 | 5.27 | 11.12 | ▅▇▆▁▂ |
| ft9_delta | 0 | 1.00 | 42.53 | 13.10 | 8.24 | 39.47 | 45.27 | 50.61 | 59.87 | ▂▁▂▇▇ |
| ft9_theta | 0 | 1.00 | 23.84 | 7.52 | 14.55 | 20.18 | 22.76 | 25.60 | 53.01 | ▇▇▁▁▁ |
| ft9_alpha | 0 | 1.00 | 16.47 | 9.49 | 0.00 | 11.08 | 16.83 | 21.96 | 37.41 | ▃▅▇▃▁ |
| ft9_low_b | 0 | 1.00 | 8.02 | 2.94 | 3.76 | 6.26 | 7.27 | 8.85 | 16.01 | ▅▇▂▂▁ |
| ft9_high_b | 0 | 1.00 | 5.18 | 3.60 | 0.31 | 3.43 | 4.26 | 6.20 | 13.12 | ▃▇▂▁▂ |
| ft9_gamma | 0 | 1.00 | 3.96 | 2.69 | 0.34 | 2.16 | 2.90 | 5.35 | 10.36 | ▆▇▃▁▃ |
| fc5_delta | 0 | 1.00 | 38.98 | 11.79 | 4.63 | 34.77 | 40.16 | 45.46 | 57.82 | ▁▁▅▇▃ |
| fc5_theta | 0 | 1.00 | 20.52 | 9.58 | 0.00 | 16.75 | 21.37 | 25.57 | 41.17 | ▂▂▇▅▂ |
| fc5_alpha | 0 | 1.00 | 19.19 | 13.15 | 1.91 | 10.72 | 16.56 | 22.72 | 52.79 | ▅▇▂▂▁ |
| fc5_low_b | 0 | 1.00 | 8.60 | 3.55 | 1.26 | 6.50 | 8.14 | 10.68 | 17.70 | ▂▇▆▃▁ |
| fc5_high_b | 0 | 1.00 | 7.03 | 4.31 | 0.00 | 4.88 | 6.72 | 8.04 | 23.14 | ▃▇▂▁▁ |
| fc5_gamma | 0 | 1.00 | 5.69 | 3.42 | 2.07 | 3.33 | 4.74 | 6.79 | 15.98 | ▇▃▂▁▁ |
| fc1_delta | 0 | 1.00 | 35.32 | 16.96 | 4.07 | 21.13 | 36.61 | 44.91 | 73.19 | ▃▂▇▃▁ |
| fc1_theta | 0 | 1.00 | 20.37 | 8.39 | 3.08 | 17.34 | 19.94 | 21.89 | 43.84 | ▂▇▇▂▁ |
| fc1_alpha | 0 | 1.00 | 19.52 | 12.59 | 1.66 | 14.17 | 17.03 | 21.44 | 66.41 | ▃▇▁▁▁ |
| fc1_low_b | 0 | 1.00 | 10.36 | 4.85 | 0.78 | 7.35 | 9.65 | 13.09 | 21.01 | ▂▇▇▅▂ |
| fc1_high_b | 0 | 1.00 | 8.36 | 5.09 | 1.10 | 4.40 | 7.08 | 10.78 | 19.34 | ▇▇▆▁▅ |
| fc1_gamma | 0 | 1.00 | 6.07 | 4.65 | 0.64 | 1.95 | 5.32 | 8.85 | 15.77 | ▇▃▃▁▂ |
| fc2_delta | 0 | 1.00 | 33.88 | 14.57 | 0.01 | 20.87 | 35.60 | 47.10 | 60.04 | ▁▇▅▇▅ |
| fc2_theta | 0 | 1.00 | 18.51 | 7.08 | 0.00 | 16.59 | 18.57 | 20.59 | 36.70 | ▁▁▇▂▁ |
| fc2_alpha | 0 | 1.00 | 21.71 | 13.81 | 1.40 | 15.15 | 17.44 | 22.28 | 55.26 | ▂▇▁▁▁ |
| fc2_low_b | 0 | 1.00 | 10.94 | 5.02 | 0.51 | 7.66 | 9.86 | 13.23 | 23.37 | ▂▇▇▃▂ |
| fc2_high_b | 0 | 1.00 | 8.04 | 5.31 | 0.32 | 3.96 | 7.65 | 10.88 | 18.79 | ▇▇▇▁▅ |
| fc2_gamma | 0 | 1.00 | 6.93 | 5.38 | 0.85 | 2.00 | 6.16 | 9.29 | 23.06 | ▇▇▁▂▁ |
| fc6_delta | 0 | 1.00 | 36.50 | 15.85 | 2.85 | 31.07 | 41.34 | 45.85 | 59.77 | ▂▂▂▇▂ |
| fc6_theta | 0 | 1.00 | 21.65 | 8.63 | 1.17 | 17.71 | 22.43 | 27.32 | 35.85 | ▂▂▇▇▅ |
| fc6_alpha | 0 | 1.00 | 20.92 | 13.97 | 1.40 | 12.46 | 18.37 | 27.44 | 60.17 | ▇▇▃▂▁ |
| fc6_low_b | 0 | 1.00 | 8.42 | 4.18 | 0.69 | 6.98 | 8.53 | 9.63 | 18.93 | ▂▅▇▁▂ |
| fc6_high_b | 0 | 1.00 | 6.90 | 4.37 | 0.52 | 4.81 | 6.28 | 8.19 | 22.41 | ▅▇▂▁▁ |
| fc6_gamma | 0 | 1.00 | 5.60 | 4.32 | 0.45 | 3.60 | 4.90 | 6.09 | 25.31 | ▇▅▁▁▁ |
| ft10_delta | 0 | 1.00 | 44.73 | 13.15 | 8.55 | 40.69 | 46.30 | 52.71 | 64.52 | ▁▂▂▇▅ |
| ft10_theta | 0 | 1.00 | 22.01 | 7.59 | 2.25 | 17.12 | 23.03 | 26.98 | 37.35 | ▁▂▇▇▁ |
| ft10_alpha | 0 | 1.00 | 18.47 | 11.65 | 0.00 | 11.56 | 16.13 | 22.50 | 48.48 | ▂▇▂▁▂ |
| ft10_low_b | 0 | 1.00 | 6.00 | 3.11 | 0.00 | 4.90 | 6.45 | 8.05 | 10.52 | ▅▁▅▇▅ |
| ft10_high_b | 0 | 1.00 | 5.20 | 2.25 | 0.48 | 3.52 | 5.26 | 6.28 | 10.56 | ▁▃▇▁▂ |
| ft10_gamma | 0 | 1.00 | 3.59 | 2.06 | 0.23 | 2.22 | 3.04 | 4.90 | 8.77 | ▃▇▃▂▁ |
| t7_delta | 0 | 1.00 | 36.08 | 16.35 | 3.49 | 24.56 | 38.09 | 46.63 | 70.19 | ▃▂▇▃▂ |
| t7_theta | 0 | 1.00 | 17.49 | 9.40 | 0.00 | 12.31 | 16.87 | 23.67 | 36.84 | ▃▅▆▇▂ |
| t7_alpha | 0 | 1.00 | 21.60 | 15.20 | 0.00 | 12.06 | 20.07 | 27.56 | 62.63 | ▆▇▃▂▁ |
| t7_low_b | 0 | 1.00 | 9.51 | 6.17 | 0.44 | 6.66 | 8.94 | 11.60 | 25.32 | ▅▇▇▂▂ |
| t7_high_b | 0 | 1.00 | 8.01 | 5.84 | 0.00 | 4.54 | 7.29 | 9.87 | 29.94 | ▇▇▁▁▁ |
| t7_gamma | 0 | 1.00 | 7.31 | 5.60 | 1.99 | 3.41 | 5.14 | 7.88 | 21.15 | ▇▅▁▁▂ |
| c3_delta | 0 | 1.00 | 35.79 | 12.65 | 8.98 | 27.79 | 36.55 | 42.92 | 62.47 | ▃▅▇▆▂ |
| c3_theta | 0 | 1.00 | 21.25 | 7.77 | 0.00 | 17.58 | 22.14 | 25.71 | 34.47 | ▂▂▆▇▆ |
| c3_alpha | 0 | 1.00 | 25.36 | 17.81 | 1.51 | 13.65 | 20.92 | 34.91 | 68.70 | ▇▇▅▁▂ |
| c3_low_b | 0 | 1.00 | 7.91 | 3.65 | 0.61 | 5.58 | 8.24 | 9.73 | 15.60 | ▂▅▇▃▂ |
| c3_high_b | 0 | 1.00 | 6.27 | 2.80 | 0.25 | 4.61 | 5.85 | 7.89 | 13.14 | ▂▇▇▃▂ |
| c3_gamma | 0 | 1.00 | 3.43 | 2.13 | 0.00 | 1.91 | 3.18 | 4.23 | 8.04 | ▆▇▇▂▃ |
| cz_delta | 0 | 1.00 | 33.90 | 17.32 | 0.01 | 24.79 | 38.64 | 45.97 | 70.40 | ▅▃▇▇▁ |
| cz_theta | 0 | 1.00 | 19.79 | 10.29 | 2.22 | 15.61 | 19.72 | 25.43 | 42.44 | ▃▅▇▂▂ |
| cz_alpha | 0 | 1.00 | 27.35 | 19.52 | 2.58 | 13.41 | 20.84 | 34.97 | 78.90 | ▇▇▃▁▂ |
| cz_low_b | 0 | 1.00 | 8.36 | 3.31 | 0.00 | 6.28 | 8.27 | 10.31 | 16.81 | ▁▇▇▅▁ |
| cz_high_b | 0 | 1.00 | 6.12 | 3.34 | 0.82 | 3.32 | 5.94 | 8.21 | 16.14 | ▇▇▇▂▁ |
| cz_gamma | 0 | 1.00 | 4.48 | 3.35 | 0.18 | 1.88 | 4.23 | 6.75 | 15.56 | ▇▅▅▁▁ |
| c4_delta | 0 | 1.00 | 35.06 | 14.47 | 5.01 | 26.25 | 36.36 | 45.18 | 59.65 | ▃▅▇▇▅ |
| c4_theta | 0 | 1.00 | 20.06 | 11.03 | 2.38 | 14.02 | 20.74 | 25.20 | 58.44 | ▃▇▃▁▁ |
| c4_alpha | 0 | 1.00 | 28.33 | 17.66 | 0.03 | 14.62 | 23.75 | 37.21 | 69.30 | ▃▇▅▂▂ |
| c4_low_b | 0 | 1.00 | 6.94 | 3.90 | 0.53 | 4.37 | 7.07 | 9.61 | 15.18 | ▅▇▇▆▂ |
| c4_high_b | 0 | 1.00 | 5.81 | 2.74 | 0.31 | 4.41 | 5.77 | 7.12 | 12.43 | ▃▃▇▂▁ |
| c4_gamma | 0 | 1.00 | 3.80 | 2.87 | 0.27 | 1.96 | 2.95 | 4.41 | 11.46 | ▇▅▁▁▂ |
| t8_delta | 0 | 1.00 | 30.75 | 20.15 | 0.01 | 7.82 | 37.19 | 41.16 | 64.56 | ▇▁▇▅▅ |
| t8_theta | 0 | 1.00 | 20.23 | 10.95 | 0.58 | 15.94 | 20.58 | 25.09 | 41.61 | ▃▃▇▂▃ |
| t8_alpha | 0 | 1.00 | 21.44 | 15.27 | 1.61 | 11.30 | 19.29 | 29.59 | 68.73 | ▇▆▃▁▁ |
| t8_low_b | 0 | 1.00 | 10.49 | 5.52 | 0.90 | 7.14 | 9.58 | 14.68 | 20.92 | ▅▇▇▇▃ |
| t8_high_b | 0 | 1.00 | 9.90 | 8.30 | 0.28 | 5.28 | 8.38 | 11.84 | 34.50 | ▇▇▂▁▁ |
| t8_gamma | 0 | 1.00 | 7.18 | 5.79 | 0.00 | 3.51 | 5.28 | 10.88 | 26.84 | ▇▃▃▁▁ |
| tp9_delta | 0 | 1.00 | 38.38 | 15.66 | 3.96 | 32.84 | 40.73 | 46.90 | 67.54 | ▃▂▇▆▃ |
| tp9_theta | 0 | 1.00 | 20.58 | 7.52 | 2.56 | 16.17 | 22.73 | 24.44 | 35.22 | ▂▂▆▇▂ |
| tp9_alpha | 0 | 1.00 | 21.29 | 15.22 | 1.27 | 11.31 | 17.93 | 26.33 | 61.27 | ▆▇▅▁▂ |
| tp9_low_b | 0 | 1.00 | 9.07 | 4.75 | 0.00 | 6.76 | 8.89 | 11.08 | 21.12 | ▂▇▇▂▁ |
| tp9_high_b | 0 | 1.00 | 6.54 | 4.46 | 0.37 | 3.89 | 6.03 | 7.37 | 19.26 | ▅▇▁▁▁ |
| tp9_gamma | 0 | 1.00 | 4.13 | 3.33 | 0.24 | 2.12 | 3.06 | 4.97 | 14.44 | ▇▅▂▁▁ |
| cp5_delta | 0 | 1.00 | 36.90 | 14.66 | 1.07 | 31.14 | 40.20 | 46.45 | 63.82 | ▂▁▅▇▁ |
| cp5_theta | 0 | 1.00 | 19.75 | 8.13 | 2.61 | 15.33 | 20.71 | 25.75 | 34.75 | ▂▅▇▅▃ |
| cp5_alpha | 0 | 1.00 | 27.63 | 16.77 | 2.04 | 16.44 | 23.64 | 33.76 | 75.27 | ▅▇▃▁▁ |
| cp5_low_b | 0 | 1.00 | 7.25 | 4.34 | 0.00 | 5.51 | 7.30 | 9.23 | 21.13 | ▃▇▅▁▁ |
| cp5_high_b | 0 | 1.00 | 5.32 | 4.07 | 0.19 | 3.48 | 4.68 | 5.93 | 21.67 | ▆▇▁▁▁ |
| cp5_gamma | 0 | 1.00 | 3.14 | 4.28 | 0.27 | 1.06 | 1.97 | 3.25 | 22.63 | ▇▁▁▁▁ |
| cp1_delta | 0 | 1.00 | 35.83 | 18.45 | 2.86 | 25.60 | 37.86 | 51.12 | 72.75 | ▅▇▇▅▂ |
| cp1_theta | 0 | 1.00 | 17.18 | 10.75 | 0.00 | 7.24 | 18.73 | 24.34 | 41.97 | ▇▅▇▅▂ |
| cp1_alpha | 0 | 1.00 | 31.85 | 21.49 | 1.79 | 15.77 | 27.59 | 44.77 | 81.29 | ▇▇▆▂▂ |
| cp1_low_b | 0 | 1.00 | 7.07 | 3.75 | 0.46 | 5.50 | 7.08 | 8.69 | 19.46 | ▃▇▅▁▁ |
| cp1_high_b | 0 | 1.00 | 4.92 | 2.50 | 0.43 | 3.79 | 4.89 | 5.74 | 12.65 | ▃▇▃▂▁ |
| cp1_gamma | 0 | 1.00 | 3.15 | 2.14 | 0.27 | 1.75 | 2.53 | 4.20 | 10.21 | ▇▆▂▁▁ |
| cp2_delta | 0 | 1.00 | 35.03 | 18.95 | 0.01 | 25.14 | 38.26 | 48.31 | 78.02 | ▅▃▇▆▁ |
| cp2_theta | 0 | 1.00 | 17.50 | 12.85 | 0.00 | 5.82 | 17.29 | 22.28 | 53.97 | ▆▇▃▁▁ |
| cp2_alpha | 0 | 1.00 | 31.99 | 20.77 | 1.41 | 18.13 | 27.67 | 40.86 | 86.68 | ▆▇▃▁▂ |
| cp2_low_b | 0 | 1.00 | 7.99 | 4.78 | 0.49 | 5.77 | 7.36 | 9.08 | 23.04 | ▃▇▂▁▁ |
| cp2_high_b | 0 | 1.00 | 4.37 | 3.20 | 0.39 | 1.82 | 3.79 | 6.20 | 12.38 | ▇▇▅▂▂ |
| cp2_gamma | 0 | 1.00 | 3.11 | 2.31 | 0.18 | 1.33 | 2.78 | 3.96 | 10.34 | ▇▇▃▁▁ |
| cp6_delta | 0 | 1.00 | 35.50 | 14.11 | 2.29 | 31.32 | 38.63 | 44.44 | 67.68 | ▂▂▇▇▁ |
| cp6_theta | 0 | 1.00 | 18.88 | 10.33 | 0.00 | 13.06 | 17.98 | 27.03 | 45.31 | ▅▇▆▆▁ |
| cp6_alpha | 0 | 1.00 | 27.66 | 16.72 | 0.03 | 16.12 | 24.96 | 40.72 | 72.59 | ▃▇▅▃▁ |
| cp6_low_b | 0 | 1.00 | 9.20 | 4.48 | 4.02 | 6.68 | 8.08 | 10.93 | 25.52 | ▇▅▁▁▁ |
| cp6_high_b | 0 | 1.00 | 5.05 | 2.10 | 1.80 | 3.70 | 4.48 | 6.71 | 10.22 | ▃▇▂▃▁ |
| cp6_gamma | 0 | 1.00 | 3.71 | 3.50 | 0.35 | 1.88 | 2.23 | 4.30 | 16.69 | ▇▂▁▁▁ |
| tp10_delta | 0 | 1.00 | 37.15 | 15.70 | 5.17 | 32.09 | 39.24 | 48.09 | 60.51 | ▃▂▆▇▅ |
| tp10_theta | 0 | 1.00 | 20.71 | 9.09 | 0.58 | 18.10 | 21.40 | 25.62 | 42.38 | ▂▁▇▂▁ |
| tp10_alpha | 0 | 1.00 | 19.86 | 13.08 | 1.23 | 11.98 | 18.47 | 24.61 | 62.18 | ▅▇▂▁▁ |
| tp10_low_b | 0 | 1.00 | 9.05 | 5.30 | 0.00 | 6.60 | 9.50 | 11.15 | 20.92 | ▃▅▇▃▁ |
| tp10_high_b | 0 | 1.00 | 7.79 | 5.59 | 0.54 | 4.57 | 6.75 | 9.34 | 32.04 | ▇▆▁▁▁ |
| tp10_gamma | 0 | 1.00 | 5.44 | 5.06 | 0.19 | 2.35 | 4.57 | 7.27 | 26.84 | ▇▃▁▁▁ |
| p7_delta | 0 | 1.00 | 37.21 | 14.06 | 0.01 | 30.13 | 42.28 | 45.73 | 57.48 | ▂▁▃▇▅ |
| p7_theta | 0 | 1.00 | 19.86 | 8.61 | 1.19 | 16.39 | 20.01 | 24.82 | 40.77 | ▂▃▇▂▁ |
| p7_alpha | 0 | 1.00 | 26.46 | 15.51 | 4.30 | 15.58 | 22.72 | 30.36 | 69.90 | ▆▇▁▂▁ |
| p7_low_b | 0 | 1.00 | 7.93 | 3.80 | 0.76 | 6.30 | 7.81 | 9.55 | 15.68 | ▂▃▇▂▂ |
| p7_high_b | 0 | 1.00 | 5.27 | 3.40 | 0.41 | 3.88 | 4.34 | 6.00 | 20.00 | ▇▆▁▁▁ |
| p7_gamma | 0 | 1.00 | 3.27 | 3.14 | 0.19 | 1.66 | 2.64 | 3.65 | 15.92 | ▇▂▁▁▁ |
| p3_delta | 0 | 1.00 | 33.10 | 15.41 | 0.01 | 26.67 | 35.09 | 42.06 | 60.95 | ▃▂▇▇▃ |
| p3_theta | 0 | 1.00 | 20.31 | 8.88 | 2.94 | 14.41 | 21.12 | 25.19 | 46.59 | ▂▇▇▂▁ |
| p3_alpha | 0 | 1.00 | 28.59 | 19.64 | 2.23 | 14.57 | 25.25 | 35.78 | 87.97 | ▇▆▃▁▁ |
| p3_low_b | 0 | 1.00 | 9.28 | 5.01 | 0.00 | 6.58 | 8.09 | 11.21 | 24.60 | ▂▇▃▁▁ |
| p3_high_b | 0 | 1.00 | 5.53 | 3.46 | 0.00 | 3.05 | 4.65 | 7.11 | 13.54 | ▃▇▅▂▂ |
| p3_gamma | 0 | 1.00 | 3.19 | 2.55 | 0.20 | 1.15 | 2.43 | 5.20 | 9.34 | ▇▅▂▂▁ |
| pz_delta | 0 | 1.00 | 37.28 | 14.65 | 3.64 | 24.95 | 39.36 | 47.77 | 58.04 | ▂▅▅▇▇ |
| pz_theta | 0 | 1.00 | 19.30 | 9.35 | 1.96 | 13.44 | 20.89 | 24.86 | 42.15 | ▃▅▇▃▁ |
| pz_alpha | 0 | 1.00 | 30.16 | 19.32 | 0.01 | 16.44 | 25.61 | 41.31 | 82.29 | ▅▇▂▂▁ |
| pz_low_b | 0 | 1.00 | 7.16 | 3.34 | 0.72 | 5.74 | 7.02 | 8.76 | 16.46 | ▃▇▇▃▁ |
| pz_high_b | 0 | 1.00 | 3.86 | 2.13 | 0.00 | 2.38 | 3.90 | 5.00 | 8.67 | ▃▅▇▂▂ |
| pz_gamma | 0 | 1.00 | 2.24 | 1.53 | 0.11 | 1.39 | 1.86 | 2.74 | 6.33 | ▅▇▂▁▁ |
| p4_delta | 0 | 1.00 | 33.42 | 18.17 | 2.08 | 17.97 | 39.79 | 46.32 | 56.61 | ▆▁▂▇▆ |
| p4_theta | 0 | 1.00 | 19.22 | 10.78 | 1.56 | 12.60 | 21.37 | 26.24 | 48.53 | ▃▃▇▁▁ |
| p4_alpha | 0 | 1.00 | 31.99 | 23.91 | 1.38 | 16.05 | 26.79 | 39.17 | 84.88 | ▇▇▅▁▃ |
| p4_low_b | 0 | 1.00 | 7.88 | 3.54 | 0.57 | 6.26 | 7.70 | 8.59 | 15.87 | ▂▃▇▁▂ |
| p4_high_b | 0 | 1.00 | 4.57 | 3.13 | 0.11 | 3.41 | 3.92 | 5.27 | 16.30 | ▃▇▂▁▁ |
| p4_gamma | 0 | 1.00 | 2.91 | 2.51 | 0.13 | 1.56 | 2.19 | 3.42 | 12.68 | ▇▃▁▁▁ |
| p8_delta | 0 | 1.00 | 33.62 | 16.00 | 3.68 | 28.00 | 37.63 | 44.18 | 58.35 | ▃▁▅▇▃ |
| p8_theta | 0 | 1.00 | 18.72 | 11.00 | 0.00 | 12.05 | 20.20 | 24.84 | 51.28 | ▃▅▇▁▁ |
| p8_alpha | 0 | 1.00 | 25.17 | 14.86 | 0.03 | 17.85 | 23.22 | 32.06 | 59.63 | ▂▇▅▂▂ |
| p8_low_b | 0 | 1.00 | 10.95 | 5.09 | 0.52 | 7.75 | 10.28 | 13.48 | 20.56 | ▂▇▇▃▃ |
| p8_high_b | 0 | 1.00 | 6.08 | 4.55 | 0.00 | 3.20 | 5.33 | 9.09 | 15.95 | ▅▇▂▃▂ |
| p8_gamma | 0 | 1.00 | 5.45 | 4.48 | 0.16 | 2.21 | 3.67 | 7.79 | 15.53 | ▇▅▂▁▃ |
| o1_delta | 0 | 1.00 | 39.37 | 12.59 | 7.11 | 33.04 | 40.21 | 45.28 | 61.49 | ▂▃▇▇▅ |
| o1_theta | 0 | 1.00 | 19.16 | 7.51 | 1.67 | 14.61 | 18.39 | 24.75 | 34.60 | ▁▃▇▆▂ |
| o1_alpha | 0 | 1.00 | 23.79 | 15.82 | 1.86 | 14.41 | 18.66 | 29.94 | 65.20 | ▅▇▂▃▁ |
| o1_low_b | 0 | 1.00 | 7.93 | 5.70 | 0.00 | 6.20 | 7.77 | 9.91 | 30.69 | ▃▇▂▁▁ |
| o1_high_b | 0 | 1.00 | 5.41 | 3.91 | 0.00 | 2.91 | 4.94 | 7.12 | 16.33 | ▇▇▂▂▁ |
| o1_gamma | 0 | 1.00 | 4.34 | 3.25 | 0.56 | 1.80 | 2.67 | 6.32 | 12.04 | ▇▂▂▂▂ |
| oz_delta | 0 | 1.00 | 39.16 | 13.83 | 1.65 | 35.24 | 38.21 | 49.13 | 60.57 | ▂▁▅▇▆ |
| oz_theta | 0 | 1.00 | 19.35 | 8.05 | 1.31 | 14.91 | 19.52 | 24.15 | 39.89 | ▁▇▇▃▁ |
| oz_alpha | 0 | 1.00 | 26.81 | 17.82 | 0.00 | 14.68 | 23.31 | 34.10 | 79.61 | ▆▇▃▂▁ |
| oz_low_b | 0 | 1.00 | 7.49 | 2.41 | 0.79 | 6.44 | 7.46 | 8.50 | 12.65 | ▁▁▇▅▂ |
| oz_high_b | 0 | 1.00 | 4.35 | 2.38 | 0.37 | 2.81 | 3.88 | 5.90 | 8.60 | ▃▇▅▂▅ |
| oz_gamma | 0 | 1.00 | 2.84 | 2.17 | 0.00 | 1.34 | 2.15 | 3.58 | 7.42 | ▅▇▁▂▂ |
| o2_delta | 0 | 1.00 | 35.87 | 15.61 | 3.79 | 31.25 | 38.24 | 45.83 | 67.36 | ▃▁▇▇▁ |
| o2_theta | 0 | 1.00 | 18.65 | 11.24 | 0.00 | 12.84 | 16.61 | 23.82 | 49.00 | ▃▇▅▁▁ |
| o2_alpha | 0 | 1.00 | 28.90 | 17.22 | 3.21 | 17.44 | 24.08 | 37.30 | 73.74 | ▅▇▅▁▂ |
| o2_low_b | 0 | 1.00 | 8.65 | 3.04 | 1.48 | 6.75 | 8.37 | 9.93 | 14.47 | ▁▃▇▂▃ |
| o2_high_b | 0 | 1.00 | 4.90 | 2.74 | 0.33 | 3.00 | 3.96 | 6.61 | 13.29 | ▃▇▂▂▁ |
| o2_gamma | 0 | 1.00 | 3.03 | 2.75 | 0.00 | 1.51 | 2.11 | 3.90 | 13.01 | ▇▃▁▁▁ |
| total_delta | 0 | 1.00 | 37.25 | 8.44 | 17.40 | 33.77 | 39.18 | 42.29 | 51.44 | ▂▁▅▇▂ |
| total_theta | 0 | 1.00 | 20.01 | 4.07 | 13.07 | 16.63 | 20.21 | 22.60 | 28.93 | ▆▆▇▃▃ |
| total_alpha | 0 | 1.00 | 23.61 | 11.45 | 10.36 | 15.25 | 22.32 | 27.26 | 53.59 | ▇▆▁▁▂ |
| total_low_beta | 0 | 1.00 | 8.44 | 1.66 | 4.95 | 7.51 | 8.25 | 9.51 | 13.20 | ▂▇▇▂▁ |
| total_high_beta | 0 | 1.00 | 6.28 | 1.93 | 2.96 | 5.23 | 6.27 | 7.18 | 10.59 | ▅▅▇▃▂ |
| total_gamma | 0 | 1.00 | 4.41 | 1.80 | 1.46 | 3.15 | 4.30 | 5.34 | 9.38 | ▅▇▅▂▁ |
| frontal_delta | 0 | 1.00 | 39.43 | 8.32 | 22.66 | 36.40 | 39.89 | 44.51 | 55.44 | ▃▁▇▇▁ |
| frontal_theta | 0 | 1.00 | 20.95 | 4.08 | 13.94 | 18.05 | 20.64 | 23.48 | 29.81 | ▃▇▇▃▂ |
| frontal_alpha | 0 | 1.00 | 19.18 | 9.68 | 8.84 | 13.13 | 15.87 | 22.40 | 44.49 | ▇▅▂▁▂ |
| frontal_low_beta | 0 | 1.00 | 8.45 | 1.61 | 5.53 | 7.49 | 8.36 | 9.12 | 12.17 | ▃▅▇▃▂ |
| frontal_high_beta | 0 | 1.00 | 6.99 | 2.15 | 3.38 | 5.05 | 7.27 | 8.38 | 10.91 | ▆▃▆▇▂ |
| frontal_gamma | 0 | 1.00 | 5.00 | 1.79 | 1.68 | 3.52 | 5.27 | 5.91 | 8.30 | ▃▆▇▅▃ |

## Table printed with `knitr::kable()`, not {gt}. Learn why at
## https://www.danieldsjoberg.com/gtsummary/articles/rmarkdown.html
## To suppress this message, include `message = FALSE` in the code chunk header.

| **Characteristic** | **N = 31** |
| --- | --- |
| subject | 117 (109, 127) |
| Unknown | 1 |
| sg_ratio | 0.57 (0.39, 0.81) |
| Unknown | 1 |
| sdnn | 64 (43, 88) |
| Unknown | 2 |
| rmssd | 36 (25, 47) |
| Unknown | 2 |
| vlf | 1,007 (484, 2,517) |
| Unknown | 2 |
| lf | 2,051 (1,141, 2,863) |
| Unknown | 2 |
| hf | 505 (273, 857) |
| Unknown | 2 |
| lf_hf_ratio | 3.71 (2.19, 6.02) |
| Unknown | 2 |
| fp1_delta | 42 (38, 50) |
| fp1_theta | 20 (16, 23) |
| fp1_alpha | 14 (11, 20) |
| fp1_low_b | 7.6 (6.5, 9.7) |
| fp1_high_b | 5.9 (3.8, 7.7) |
| fp1_gamma | 4.2 (2.6, 6.8) |
| fp2_delta | 41 (29, 49) |
| fp2_theta | 21 (15, 23) |
| fp2_alpha | 17 (13, 20) |
| fp2_low_b | 7.75 (6.47, 10.36) |
| fp2_high_b | 6.3 (4.0, 8.9) |
| fp2_gamma | 3.9 (2.6, 6.5) |
| f7_delta | 47 (39, 50) |
| f7_theta | 21.1 (17.0, 23.6) |
| f7_alpha | 16 (12, 22) |
| f7_low_b | 7.68 (6.47, 8.71) |
| f7_high_b | 5.8 (4.5, 7.4) |
| f7_gamma | 3.53 (0.95, 4.87) |
| f3_delta | 39 (32, 49) |
| f3_theta | 21 (18, 25) |
| f3_alpha | 16 (13, 20) |
| f3_low_b | 8.1 (7.0, 11.5) |
| f3_high_b | 7.1 (5.5, 11.4) |
| f3_gamma | 4.4 (2.6, 6.6) |
| fz_delta | 42 (35, 48) |
| fz_theta | 22 (16, 27) |
| fz_alpha | 16 (13, 21) |
| fz_low_b | 7.94 (6.38, 9.53) |
| fz_high_b | 6.0 (3.8, 7.6) |
| fz_gamma | 3.70 (1.63, 5.38) |
| f4_delta | 36 (23, 45) |
| f4_theta | 22 (19, 31) |
| f4_alpha | 16 (10, 24) |
| f4_low_b | 9.2 (7.5, 12.6) |
| f4_high_b | 8.2 (4.9, 11.1) |
| f4_gamma | 5.4 (2.8, 10.5) |
| f8_delta | 47 (38, 55) |
| f8_theta | 21 (17, 23) |
| f8_alpha | 15 (12, 23) |
| f8_low_b | 6.9 (5.1, 8.5) |
| f8_high_b | 5.6 (4.2, 7.5) |
| f8_gamma | 3.80 (2.02, 5.27) |
| ft9_delta | 45 (39, 51) |
| ft9_theta | 23 (20, 26) |
| ft9_alpha | 17 (11, 22) |
| ft9_low_b | 7.27 (6.26, 8.85) |
| ft9_high_b | 4.3 (3.4, 6.2) |
| ft9_gamma | 2.90 (2.16, 5.35) |
| fc5_delta | 40 (35, 45) |
| fc5_theta | 21 (17, 26) |
| fc5_alpha | 17 (11, 23) |
| fc5_low_b | 8.1 (6.5, 10.7) |
| fc5_high_b | 6.7 (4.9, 8.0) |
| fc5_gamma | 4.7 (3.3, 6.8) |
| fc1_delta | 37 (21, 45) |
| fc1_theta | 20 (17, 22) |
| fc1_alpha | 17 (14, 21) |
| fc1_low_b | 9.7 (7.3, 13.1) |
| fc1_high_b | 7.1 (4.4, 10.8) |
| fc1_gamma | 5.3 (1.9, 8.8) |
| fc2_delta | 36 (21, 47) |
| fc2_theta | 19 (17, 21) |
| fc2_alpha | 17 (15, 22) |
| fc2_low_b | 9.9 (7.7, 13.2) |
| fc2_high_b | 7.7 (4.0, 10.9) |
| fc2_gamma | 6.2 (2.0, 9.3) |
| fc6_delta | 41 (31, 46) |
| fc6_theta | 22 (18, 27) |
| fc6_alpha | 18 (12, 27) |
| fc6_low_b | 8.5 (7.0, 9.6) |
| fc6_high_b | 6.3 (4.8, 8.2) |
| fc6_gamma | 4.90 (3.60, 6.09) |
| ft10_delta | 46 (41, 53) |
| ft10_theta | 23 (17, 27) |
| ft10_alpha | 16 (12, 22) |
| ft10_low_b | 6.45 (4.90, 8.05) |
| ft10_high_b | 5.26 (3.52, 6.28) |
| ft10_gamma | 3.04 (2.22, 4.90) |
| t7_delta | 38 (25, 47) |
| t7_theta | 17 (12, 24) |
| t7_alpha | 20 (12, 28) |
| t7_low_b | 9 (7, 12) |
| t7_high_b | 7.3 (4.5, 9.9) |
| t7_gamma | 5.1 (3.4, 7.9) |
| c3_delta | 37 (28, 43) |
| c3_theta | 22 (18, 26) |
| c3_alpha | 21 (14, 35) |
| c3_low_b | 8.2 (5.6, 9.7) |
| c3_high_b | 5.85 (4.61, 7.89) |
| c3_gamma | 3.18 (1.91, 4.23) |
| cz_delta | 39 (25, 46) |
| cz_theta | 20 (16, 25) |
| cz_alpha | 21 (13, 35) |
| cz_low_b | 8.27 (6.28, 10.31) |
| cz_high_b | 5.94 (3.32, 8.21) |
| cz_gamma | 4.23 (1.88, 6.75) |
| c4_delta | 36 (26, 45) |
| c4_theta | 21 (14, 25) |
| c4_alpha | 24 (15, 37) |
| c4_low_b | 7.1 (4.4, 9.6) |
| c4_high_b | 5.77 (4.41, 7.12) |
| c4_gamma | 2.95 (1.96, 4.41) |
| t8_delta | 37 (8, 41) |
| t8_theta | 21 (16, 25) |
| t8_alpha | 19 (11, 30) |
| t8_low_b | 9.6 (7.1, 14.7) |
| t8_high_b | 8 (5, 12) |
| t8_gamma | 5.3 (3.5, 10.9) |
| tp9_delta | 41 (33, 47) |
| tp9_theta | 23 (16, 24) |
| tp9_alpha | 18 (11, 26) |
| tp9_low_b | 8.9 (6.8, 11.1) |
| tp9_high_b | 6.0 (3.9, 7.4) |
| tp9_gamma | 3.1 (2.1, 5.0) |
| cp5_delta | 40 (31, 46) |
| cp5_theta | 21 (15, 26) |
| cp5_alpha | 24 (16, 34) |
| cp5_low_b | 7.3 (5.5, 9.2) |
| cp5_high_b | 4.7 (3.5, 5.9) |
| cp5_gamma | 1.97 (1.06, 3.25) |
| cp1_delta | 38 (26, 51) |
| cp1_theta | 19 (7, 24) |
| cp1_alpha | 28 (16, 45) |
| cp1_low_b | 7.1 (5.5, 8.7) |
| cp1_high_b | 4.89 (3.79, 5.74) |
| cp1_gamma | 2.53 (1.75, 4.20) |
| cp2_delta | 38 (25, 48) |
| cp2_theta | 17 (6, 22) |
| cp2_alpha | 28 (18, 41) |
| cp2_low_b | 7.4 (5.8, 9.1) |
| cp2_high_b | 3.79 (1.82, 6.20) |
| cp2_gamma | 2.78 (1.33, 3.96) |
| cp6_delta | 39 (31, 44) |
| cp6_theta | 18 (13, 27) |
| cp6_alpha | 25 (16, 41) |
| cp6_low_b | 8.1 (6.7, 10.9) |
| cp6_high_b | 4.48 (3.70, 6.71) |
| cp6_gamma | 2.23 (1.88, 4.30) |
| tp10_delta | 39 (32, 48) |
| tp10_theta | 21 (18, 26) |
| tp10_alpha | 18 (12, 25) |
| tp10_low_b | 9.5 (6.6, 11.2) |
| tp10_high_b | 6.7 (4.6, 9.3) |
| tp10_gamma | 4.6 (2.4, 7.3) |
| p7_delta | 42 (30, 46) |
| p7_theta | 20 (16, 25) |
| p7_alpha | 23 (16, 30) |
| p7_low_b | 7.8 (6.3, 9.5) |
| p7_high_b | 4.34 (3.88, 6.00) |
| p7_gamma | 2.64 (1.66, 3.65) |
| p3_delta | 35 (27, 42) |
| p3_theta | 21 (14, 25) |
| p3_alpha | 25 (15, 36) |
| p3_low_b | 8.1 (6.6, 11.2) |
| p3_high_b | 4.6 (3.0, 7.1) |
| p3_gamma | 2.43 (1.15, 5.20) |
| pz_delta | 39 (25, 48) |
| pz_theta | 21 (13, 25) |
| pz_alpha | 26 (16, 41) |
| pz_low_b | 7.0 (5.7, 8.8) |
| pz_high_b | 3.90 (2.38, 5.00) |
| pz_gamma | 1.86 (1.39, 2.74) |
| p4_delta | 40 (18, 46) |
| p4_theta | 21 (13, 26) |
| p4_alpha | 27 (16, 39) |
| p4_low_b | 7.7 (6.3, 8.6) |
| p4_high_b | 3.92 (3.41, 5.27) |
| p4_gamma | 2.19 (1.56, 3.42) |
| p8_delta | 38 (28, 44) |
| p8_theta | 20 (12, 25) |
| p8_alpha | 23 (18, 32) |
| p8_low_b | 10.3 (7.7, 13.5) |
| p8_high_b | 5.3 (3.2, 9.1) |
| p8_gamma | 3.7 (2.2, 7.8) |
| o1_delta | 40 (33, 45) |
| o1_theta | 18 (15, 25) |
| o1_alpha | 19 (14, 30) |
| o1_low_b | 7.8 (6.2, 9.9) |
| o1_high_b | 4.9 (2.9, 7.1) |
| o1_gamma | 2.67 (1.80, 6.32) |
| oz_delta | 38 (35, 49) |
| oz_theta | 20 (15, 24) |
| oz_alpha | 23 (15, 34) |
| oz_low_b | 7.46 (6.44, 8.50) |
| oz_high_b | 3.88 (2.81, 5.90) |
| oz_gamma | 2.15 (1.34, 3.58) |
| o2_delta | 38 (31, 46) |
| o2_theta | 17 (13, 24) |
| o2_alpha | 24 (17, 37) |
| o2_low_b | 8.37 (6.75, 9.93) |
| o2_high_b | 3.96 (3.00, 6.61) |
| o2_gamma | 2.11 (1.51, 3.90) |
| total_delta | 39 (34, 42) |
| total_theta | 20.2 (16.6, 22.6) |
| total_alpha | 22 (15, 27) |
| total_low_beta | 8.25 (7.51, 9.51) |
| total_high_beta | 6.27 (5.23, 7.18) |
| total_gamma | 4.30 (3.15, 5.34) |
| frontal_delta | 40 (36, 45) |
| frontal_theta | 20.6 (18.1, 23.5) |
| frontal_alpha | 16 (13, 22) |
| frontal_low_beta | 8.36 (7.49, 9.12) |
| frontal_high_beta | 7.27 (5.05, 8.38) |
| frontal_gamma | 5.27 (3.52, 5.91) |

# Feat. Selection

Ho cosiderato tutte le possibili variabili, anche quelle calcolate tipo le “frontal_”

## SDNN

## Boruta performed 999 iterations in 7.638724 secs.
## 4 attributes confirmed important: f7_gamma, fc2_gamma, o2_low_b,
## t8_alpha;
## 199 attributes confirmed unimportant: c3_alpha, c3_delta, c3_gamma,
## c3_high_b, c3_low_b and 194 more;
## 1 tentative attributes left: fz_alpha;
## Boruta performed 999 iterations in 7.638724 secs.
## Tentatives roughfixed over the last 999 iterations.
## 4 attributes confirmed important: f7_gamma, fc2_gamma, o2_low_b,
## t8_alpha;
## 200 attributes confirmed unimportant: c3_alpha, c3_delta, c3_gamma,
## c3_high_b, c3_low_b and 195 more;


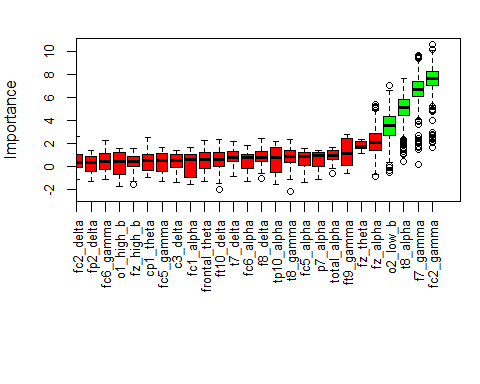


## Boruta performed 718 iterations in 5.270082 secs.
## 5 attributes confirmed important: f7_gamma, fc2_gamma, ft10_delta,
## fz_theta, t8_alpha;
## 199 attributes confirmed unimportant: c3_alpha, c3_delta, c3_gamma,
## c3_high_b, c3_low_b and 194 more;
## Boruta performed 718 iterations in 5.270082 secs.
## 5 attributes confirmed important: f7_gamma, fc2_gamma, ft10_delta,
## fz_theta, t8_alpha;
## 199 attributes confirmed unimportant: c3_alpha, c3_delta, c3_gamma,
## c3_high_b, c3_low_b and 194 more;


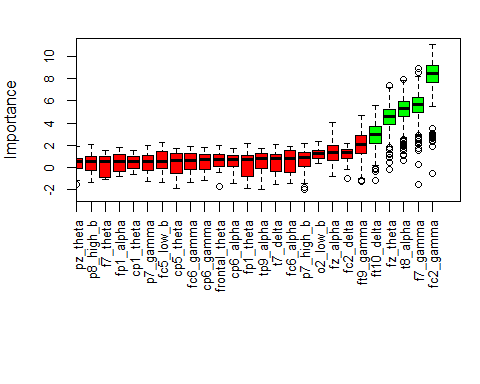


## Boruta performed 85 iterations in 0.782974 secs.
## 3 attributes confirmed important: f7_gamma, fc2_gamma, t8_alpha;
## 201 attributes confirmed unimportant: c3_alpha, c3_delta, c3_gamma,
## c3_high_b, c3_low_b and 196 more;
## Boruta performed 85 iterations in 0.782974 secs.
## 3 attributes confirmed important: f7_gamma, fc2_gamma, t8_alpha;
## 201 attributes confirmed unimportant: c3_alpha, c3_delta, c3_gamma,
## c3_high_b, c3_low_b and 196 more;


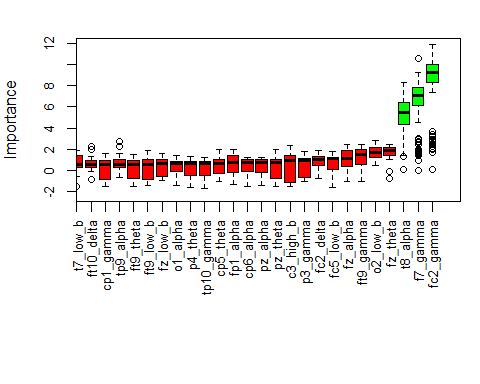


## Boruta performed 720 iterations in 5.176653 secs.
## 4 attributes confirmed important: f7_gamma, fc2_gamma, fz_theta,
## o2_low_b;
## 200 attributes confirmed unimportant: c3_alpha, c3_delta, c3_gamma,
## c3_high_b, c3_low_b and 195 more;
## Boruta performed 720 iterations in 5.176653 secs.
## 4 attributes confirmed important: f7_gamma, fc2_gamma, fz_theta,
## o2_low_b;
## 200 attributes confirmed unimportant: c3_alpha, c3_delta, c3_gamma,
## c3_high_b, c3_low_b and 195 more;


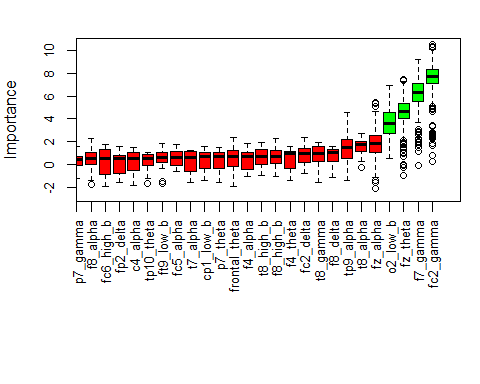


## Boruta performed 246 iterations in 2.020392 secs.
## 4 attributes confirmed important: f7_gamma, fc2_gamma, o2_low_b,
## t8_alpha;
## 200 attributes confirmed unimportant: c3_alpha, c3_delta, c3_gamma,
## c3_high_b, c3_low_b and 195 more;
## Boruta performed 246 iterations in 2.020392 secs.
## 4 attributes confirmed important: f7_gamma, fc2_gamma, o2_low_b,
## t8_alpha;
## 200 attributes confirmed unimportant: c3_alpha, c3_delta, c3_gamma,
## c3_high_b, c3_low_b and 195 more;


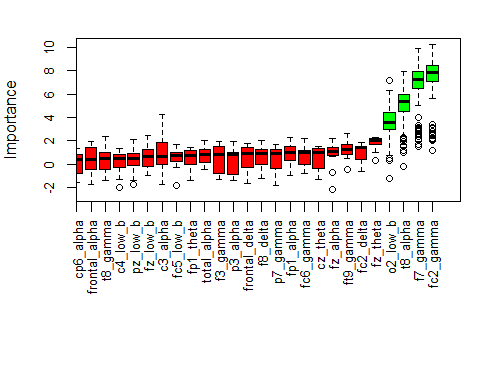
{=openxml} <w:p> <w:pPr> <w:spacing w:before="0" w:after="60"/> <w:keepNext/> <w:jc w:val="start"/> <w:pStyle w:val="caption"/> </w:pPr> <w:r> <w:rPr> <w:rFonts w:ascii="Calibri" w:hAnsi="Calibri"/> <w:sz w:val="24"/> </w:rPr> <w:t xml:space="preserve">Table </w:t> </w:r> <w:r> <w:fldChar w:fldCharType="begin" w:dirty="true"/> </w:r> <w:r> <w:instrText xml:space="preserve" w:dirty="true"> SEQ Table \* ARABIC </w:instrText> </w:r> <w:r> <w:fldChar w:fldCharType="separate" w:dirty="true"/> </w:r> <w:r> <w:rPr> <w:noProof/> <w:rFonts w:ascii="Calibri" w:hAnsi="Calibri"/> <w:sz w:val="24"/> </w:rPr> <w:t xml:space="default">1</w:t> </w:r> <w:r> <w:fldChar w:fldCharType="end" w:dirty="true"/> </w:r> <w:r> <w:rPr> <w:rFonts w:ascii="Calibri" w:hAnsi="Calibri"/> <w:sz w:val="24"/> </w:rPr> <w:t xml:space="preserve">: </w:t> </w:r> <w:r> <w:rPr> <w:b w:val="true"/> <w:rFonts w:ascii="Calibri" w:hAnsi="Calibri"/> <w:sz w:val="24"/> <w:color w:val="333333"/> </w:rPr> <w:t xml:space="preserve">Common important variables</w:t> </w:r> </w:p><w:p> <w:pPr> <w:spacing w:before="0" w:after="60"/> <w:keepNext/> <w:jc w:val="start"/> <w:pStyle w:val="caption"/> </w:pPr> <w:r> <w:rPr> <w:i/> <w:rFonts w:ascii="Calibri" w:hAnsi="Calibri"/> <w:sz w:val="20"/> <w:color w:val="333333"/> </w:rPr> <w:t xml:space="preserve">Boruta iteration on 5 seeds</w:t> </w:r> </w:p><w:tbl xmlns:w="http://schemas.openxmlformats.org/wordprocessingml/2006/main" xmlns:wp="http://schemas.openxmlformats.org/drawingml/2006/wordprocessingDrawing" xmlns:r="http://schemas.openxmlformats.org/officeDocument/2006/relationships" xmlns:w14="http://schemas.microsoft.com/office/word/2010/wordml"><w:tblPr><w:tblCellMar><w:top w:w="0" w:type="dxa"></w:top><w:bottom w:w="0" w:type="dxa"></w:bottom><w:start w:w="60" w:type="dxa"></w:start><w:end w:w="60" w:type="dxa"></w:end></w:tblCellMar><w:tblW w:type="auto" w:w="0"></w:tblW><w:tblLook w:firstRow="0" w:lastRow="0" w:firstColumn="0" w:lastColumn="0" w:noHBand="0" w:noVBand="0"></w:tblLook><w:jc w:val="center"></w:jc></w:tblPr><w:tr><w:trPr><w:cantSplit></w:cantSplit><w:tblHeader></w:tblHeader></w:trPr><w:tc><w:tcPr><w:tcBorders><w:top w:val="single" w:sz="16" w:space="0" w:color="D3D3D3"></w:top><w:bottom w:val="single" w:sz="16" w:space="0" w:color="D3D3D3"></w:bottom><w:start w:val="single" w:space="0" w:color="D3D3D3"></w:start></w:tcBorders></w:tcPr><w:p> <w:pPr> <w:spacing w:before="0" w:after="60"/> <w:keepNext/> <w:jc w:val="start"/> </w:pPr> <w:r> <w:rPr> <w:b w:val="true"/> <w:rFonts w:ascii="Calibri" w:hAnsi="Calibri"/> <w:sz w:val="20"/> </w:rPr> <w:t xml:space="preserve">Variable</w:t> </w:r> </w:p></w:tc><w:tc><w:tcPr><w:tcBorders><w:top w:val="single" w:sz="16" w:space="0" w:color="D3D3D3"></w:top><w:bottom w:val="single" w:sz="16" w:space="0" w:color="D3D3D3"></w:bottom></w:tcBorders></w:tcPr><w:p> <w:pPr> <w:spacing w:before="0" w:after="60"/> <w:keepNext/> <w:jc w:val="end"/> </w:pPr> <w:r> <w:rPr> <w:b w:val="true"/> <w:rFonts w:ascii="Calibri" w:hAnsi="Calibri"/> <w:sz w:val="20"/> </w:rPr> <w:t xml:space="preserve">MeanImp 1</w:t> </w:r> </w:p></w:tc><w:tc><w:tcPr><w:tcBorders><w:top w:val="single" w:sz="16" w:space="0" w:color="D3D3D3"></w:top><w:bottom w:val="single" w:sz="16" w:space="0" w:color="D3D3D3"></w:bottom></w:tcBorders></w:tcPr><w:p> <w:pPr> <w:spacing w:before="0" w:after="60"/> <w:keepNext/> <w:jc w:val="end"/> </w:pPr> <w:r> <w:rPr> <w:b w:val="true"/> <w:rFonts w:ascii="Calibri" w:hAnsi="Calibri"/> <w:sz w:val="20"/> </w:rPr> <w:t xml:space="preserve">MeanImp 2</w:t> </w:r> </w:p></w:tc><w:tc><w:tcPr><w:tcBorders><w:top w:val="single" w:sz="16" w:space="0" w:color="D3D3D3"></w:top><w:bottom w:val="single" w:sz="16" w:space="0" w:color="D3D3D3"></w:bottom></w:tcBorders></w:tcPr><w:p> <w:pPr> <w:spacing w:before="0" w:after="60"/> <w:keepNext/> <w:jc w:val="end"/> </w:pPr> <w:r> <w:rPr> <w:b w:val="true"/> <w:rFonts w:ascii="Calibri" w:hAnsi="Calibri"/> <w:sz w:val="20"/> </w:rPr> <w:t xml:space="preserve">MeanImp 3</w:t> </w:r> </w:p></w:tc><w:tc><w:tcPr><w:tcBorders><w:top w:val="single" w:sz="16" w:space="0" w:color="D3D3D3"></w:top><w:bottom w:val="single" w:sz="16" w:space="0" w:color="D3D3D3"></w:bottom></w:tcBorders></w:tcPr><w:p> <w:pPr> <w:spacing w:before="0" w:after="60"/> <w:keepNext/> <w:jc w:val="end"/> </w:pPr> <w:r> <w:rPr> <w:b w:val="true"/> <w:rFonts w:ascii="Calibri" w:hAnsi="Calibri"/> <w:sz w:val="20"/> </w:rPr> <w:t xml:space="preserve">MeanImp 4</w:t> </w:r> </w:p></w:tc><w:tc><w:tcPr><w:tcBorders><w:top w:val="single" w:sz="16" w:space="0" w:color="D3D3D3"></w:top><w:bottom w:val="single" w:sz="16" w:space="0" w:color="D3D3D3"></w:bottom><w:end w:val="single" w:space="0" w:color="D3D3D3"></w:end></w:tcBorders></w:tcPr><w:p> <w:pPr> <w:spacing w:before="0" w:after="60"/> <w:keepNext/> <w:jc w:val="end"/> </w:pPr> <w:r> <w:rPr> <w:b w:val="true"/> <w:rFonts w:ascii="Calibri" w:hAnsi="Calibri"/> <w:sz w:val="20"/> </w:rPr> <w:t xml:space="preserve">MeanImp 5</w:t> </w:r> </w:p></w:tc></w:tr><w:tr><w:trPr><w:cantSplit></w:cantSplit></w:trPr><w:tc><w:tcPr><w:tcBorders><w:top w:val="single" w:space="0" w:color="D3D3D3"></w:top><w:bottom w:val="single" w:space="0" w:color="D3D3D3"></w:bottom><w:start w:val="single" w:space="0" w:color="D3D3D3"></w:start><w:end w:val="single" w:space="0" w:color="D3D3D3"></w:end></w:tcBorders></w:tcPr><w:p> <w:pPr> <w:spacing w:before="0" w:after="60"/> <w:keepNext/> <w:jc w:val="start"/> </w:pPr> <w:r> <w:rPr> <w:rFonts w:ascii="Calibri" w:hAnsi="Calibri"/> <w:sz w:val="20"/> </w:rPr> <w:t xml:space="default">fc2_gamma</w:t> </w:r> </w:p></w:tc><w:tc><w:tcPr><w:tcBorders><w:top w:val="single" w:space="0" w:color="D3D3D3"></w:top><w:bottom w:val="single" w:space="0" w:color="D3D3D3"></w:bottom><w:start w:val="single" w:space="0" w:color="D3D3D3"></w:start><w:end w:val="single" w:space="0" w:color="D3D3D3"></w:end></w:tcBorders></w:tcPr><w:p> <w:pPr> <w:spacing w:before="0" w:after="60"/> <w:keepNext/> <w:jc w:val="end"/> </w:pPr> <w:r> <w:rPr> <w:rFonts w:ascii="Calibri" w:hAnsi="Calibri"/> <w:sz w:val="20"/> </w:rPr> <w:t xml:space="default">7.584013</w:t> </w:r> </w:p></w:tc><w:tc><w:tcPr><w:tcBorders><w:top w:val="single" w:space="0" w:color="D3D3D3"></w:top><w:bottom w:val="single" w:space="0" w:color="D3D3D3"></w:bottom><w:start w:val="single" w:space="0" w:color="D3D3D3"></w:start><w:end w:val="single" w:space="0" w:color="D3D3D3"></w:end></w:tcBorders></w:tcPr><w:p> <w:pPr> <w:spacing w:before="0" w:after="60"/> <w:keepNext/> <w:jc w:val="end"/> </w:pPr> <w:r> <w:rPr> <w:rFonts w:ascii="Calibri" w:hAnsi="Calibri"/> <w:sz w:val="20"/> </w:rPr> <w:t xml:space="default">8.340798</w:t> </w:r> </w:p></w:tc><w:tc><w:tcPr><w:tcBorders><w:top w:val="single" w:space="0" w:color="D3D3D3"></w:top><w:bottom w:val="single" w:space="0" w:color="D3D3D3"></w:bottom><w:start w:val="single" w:space="0" w:color="D3D3D3"></w:start><w:end w:val="single" w:space="0" w:color="D3D3D3"></w:end></w:tcBorders></w:tcPr><w:p> <w:pPr> <w:spacing w:before="0" w:after="60"/> <w:keepNext/> <w:jc w:val="end"/> </w:pPr> <w:r> <w:rPr> <w:rFonts w:ascii="Calibri" w:hAnsi="Calibri"/> <w:sz w:val="20"/> </w:rPr> <w:t xml:space="default">8.243723</w:t> </w:r> </w:p></w:tc><w:tc><w:tcPr><w:tcBorders><w:top w:val="single" w:space="0" w:color="D3D3D3"></w:top><w:bottom w:val="single" w:space="0" w:color="D3D3D3"></w:bottom><w:start w:val="single" w:space="0" w:color="D3D3D3"></w:start><w:end w:val="single" w:space="0" w:color="D3D3D3"></w:end></w:tcBorders></w:tcPr><w:p> <w:pPr> <w:spacing w:before="0" w:after="60"/> <w:keepNext/> <w:jc w:val="end"/> </w:pPr> <w:r> <w:rPr> <w:rFonts w:ascii="Calibri" w:hAnsi="Calibri"/> <w:sz w:val="20"/> </w:rPr> <w:t xml:space="default">7.575690</w:t> </w:r> </w:p></w:tc><w:tc><w:tcPr><w:tcBorders><w:top w:val="single" w:space="0" w:color="D3D3D3"></w:top><w:bottom w:val="single" w:space="0" w:color="D3D3D3"></w:bottom><w:start w:val="single" w:space="0" w:color="D3D3D3"></w:start><w:end w:val="single" w:space="0" w:color="D3D3D3"></w:end></w:tcBorders></w:tcPr><w:p> <w:pPr> <w:spacing w:before="0" w:after="60"/> <w:keepNext/> <w:jc w:val="end"/> </w:pPr> <w:r> <w:rPr> <w:rFonts w:ascii="Calibri" w:hAnsi="Calibri"/> <w:sz w:val="20"/> </w:rPr> <w:t xml:space="default">7.560806</w:t> </w:r> </w:p></w:tc></w:tr> <w:tr><w:trPr><w:cantSplit></w:cantSplit></w:trPr><w:tc><w:tcPr><w:tcBorders><w:top w:val="single" w:space="0" w:color="D3D3D3"></w:top><w:bottom w:val="single" w:space="0" w:color="D3D3D3"></w:bottom><w:start w:val="single" w:space="0" w:color="D3D3D3"></w:start><w:end w:val="single" w:space="0" w:color="D3D3D3"></w:end></w:tcBorders></w:tcPr><w:p> <w:pPr> <w:spacing w:before="0" w:after="60"/> <w:keepNext/> <w:jc w:val="start"/> </w:pPr> <w:r> <w:rPr> <w:rFonts w:ascii="Calibri" w:hAnsi="Calibri"/> <w:sz w:val="20"/> </w:rPr> <w:t xml:space="default">f7_gamma</w:t> </w:r> </w:p></w:tc><w:tc><w:tcPr><w:tcBorders><w:top w:val="single" w:space="0" w:color="D3D3D3"></w:top><w:bottom w:val="single" w:space="0" w:color="D3D3D3"></w:bottom><w:start w:val="single" w:space="0" w:color="D3D3D3"></w:start><w:end w:val="single" w:space="0" w:color="D3D3D3"></w:end></w:tcBorders></w:tcPr><w:p> <w:pPr> <w:spacing w:before="0" w:after="60"/> <w:keepNext/> <w:jc w:val="end"/> </w:pPr> <w:r> <w:rPr> <w:rFonts w:ascii="Calibri" w:hAnsi="Calibri"/> <w:sz w:val="20"/> </w:rPr> <w:t xml:space="default">6.687871</w:t> </w:r> </w:p></w:tc><w:tc><w:tcPr><w:tcBorders><w:top w:val="single" w:space="0" w:color="D3D3D3"></w:top><w:bottom w:val="single" w:space="0" w:color="D3D3D3"></w:bottom><w:start w:val="single" w:space="0" w:color="D3D3D3"></w:start><w:end w:val="single" w:space="0" w:color="D3D3D3"></w:end></w:tcBorders></w:tcPr><w:p> <w:pPr> <w:spacing w:before="0" w:after="60"/> <w:keepNext/> <w:jc w:val="end"/> </w:pPr> <w:r> <w:rPr> <w:rFonts w:ascii="Calibri" w:hAnsi="Calibri"/> <w:sz w:val="20"/> </w:rPr> <w:t xml:space="default">5.592509</w:t> </w:r> </w:p></w:tc><w:tc><w:tcPr><w:tcBorders><w:top w:val="single" w:space="0" w:color="D3D3D3"></w:top><w:bottom w:val="single" w:space="0" w:color="D3D3D3"></w:bottom><w:start w:val="single" w:space="0" w:color="D3D3D3"></w:start><w:end w:val="single" w:space="0" w:color="D3D3D3"></w:end></w:tcBorders></w:tcPr><w:p> <w:pPr> <w:spacing w:before="0" w:after="60"/> <w:keepNext/> <w:jc w:val="end"/> </w:pPr> <w:r> <w:rPr> <w:rFonts w:ascii="Calibri" w:hAnsi="Calibri"/> <w:sz w:val="20"/> </w:rPr> <w:t xml:space="default">6.408179</w:t> </w:r> </w:p></w:tc><w:tc><w:tcPr><w:tcBorders><w:top w:val="single" w:space="0" w:color="D3D3D3"></w:top><w:bottom w:val="single" w:space="0" w:color="D3D3D3"></w:bottom><w:start w:val="single" w:space="0" w:color="D3D3D3"></w:start><w:end w:val="single" w:space="0" w:color="D3D3D3"></w:end></w:tcBorders></w:tcPr><w:p> <w:pPr> <w:spacing w:before="0" w:after="60"/> <w:keepNext/> <w:jc w:val="end"/> </w:pPr> <w:r> <w:rPr> <w:rFonts w:ascii="Calibri" w:hAnsi="Calibri"/> <w:sz w:val="20"/> </w:rPr> <w:t xml:space="default">6.206432</w:t> </w:r> </w:p></w:tc><w:tc><w:tcPr><w:tcBorders><w:top w:val="single" w:space="0" w:color="D3D3D3"></w:top><w:bottom w:val="single" w:space="0" w:color="D3D3D3"></w:bottom><w:start w:val="single" w:space="0" w:color="D3D3D3"></w:start><w:end w:val="single" w:space="0" w:color="D3D3D3"></w:end></w:tcBorders></w:tcPr><w:p> <w:pPr> <w:spacing w:before="0" w:after="60"/> <w:keepNext/> <w:jc w:val="end"/> </w:pPr> <w:r> <w:rPr> <w:rFonts w:ascii="Calibri" w:hAnsi="Calibri"/> <w:sz w:val="20"/> </w:rPr> <w:t xml:space="default">7.058809</w:t> </w:r> </w:p></w:tc></w:tr></w:tbl>

## RMSSD

## Boruta performed 489 iterations in 3.758215 secs.
## 3 attributes confirmed important: fc2_gamma, fc2_low_b, fc5_theta;
## 201 attributes confirmed unimportant: c3_alpha, c3_delta, c3_gamma,
## c3_high_b, c3_low_b and 196 more;
## Boruta performed 489 iterations in 3.758215 secs.
## 3 attributes confirmed important: fc2_gamma, fc2_low_b, fc5_theta;
## 201 attributes confirmed unimportant: c3_alpha, c3_delta, c3_gamma,
## c3_high_b, c3_low_b and 196 more;


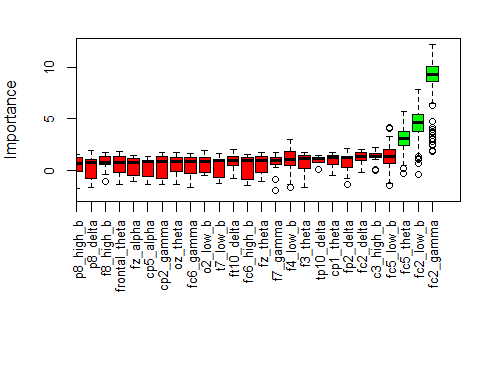


## Boruta performed 999 iterations in 7.477905 secs.
## 3 attributes confirmed important: c3_high_b, fc2_gamma, fc2_low_b;
## 200 attributes confirmed unimportant: c3_alpha, c3_delta, c3_gamma,
## c3_low_b, c3_theta and 195 more;
## 1 tentative attributes left: f3_theta;
## Boruta performed 999 iterations in 7.477905 secs.
## Tentatives roughfixed over the last 999 iterations.
## 3 attributes confirmed important: c3_high_b, fc2_gamma, fc2_low_b;
## 201 attributes confirmed unimportant: c3_alpha, c3_delta, c3_gamma,
## c3_low_b, c3_theta and 196 more;


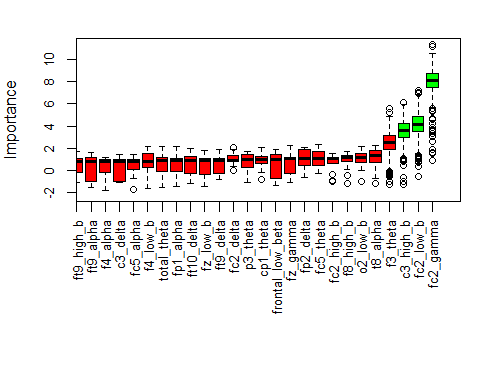


## Boruta performed 95 iterations in 0.8640399 secs.
## 2 attributes confirmed important: fc2_gamma, fc2_low_b;
## 202 attributes confirmed unimportant: c3_alpha, c3_delta, c3_gamma,
## c3_high_b, c3_low_b and 197 more;
## Boruta performed 95 iterations in 0.8640399 secs.
## 2 attributes confirmed important: fc2_gamma, fc2_low_b;
## 202 attributes confirmed unimportant: c3_alpha, c3_delta, c3_gamma,
## c3_high_b, c3_low_b and 197 more;


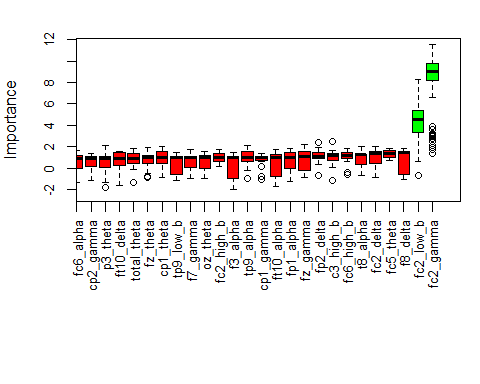


## Boruta performed 33 iterations in 0.424279 secs.
## 1 attributes confirmed important: fc2_gamma;
## 203 attributes confirmed unimportant: c3_alpha, c3_delta, c3_gamma,
## c3_high_b, c3_low_b and 198 more;
## Boruta performed 33 iterations in 0.424279 secs.
## 1 attributes confirmed important: fc2_gamma;
## 203 attributes confirmed unimportant: c3_alpha, c3_delta, c3_gamma,
## c3_high_b, c3_low_b and 198 more;


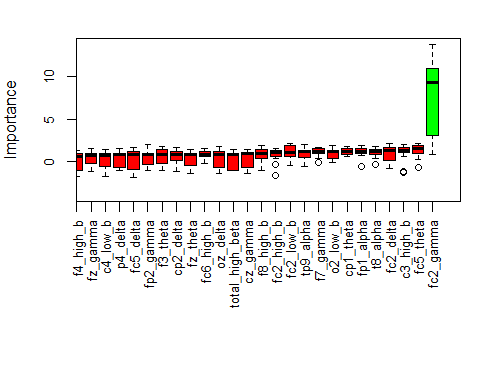


## Boruta performed 503 iterations in 3.822123 secs.
## 3 attributes confirmed important: fc2_gamma, fc2_low_b, fc5_theta;
## 201 attributes confirmed unimportant: c3_alpha, c3_delta, c3_gamma,
## c3_high_b, c3_low_b and 196 more;
## Boruta performed 503 iterations in 3.822123 secs.
## 3 attributes confirmed important: fc2_gamma, fc2_low_b, fc5_theta;
## 201 attributes confirmed unimportant: c3_alpha, c3_delta, c3_gamma,
## c3_high_b, c3_low_b and 196 more;


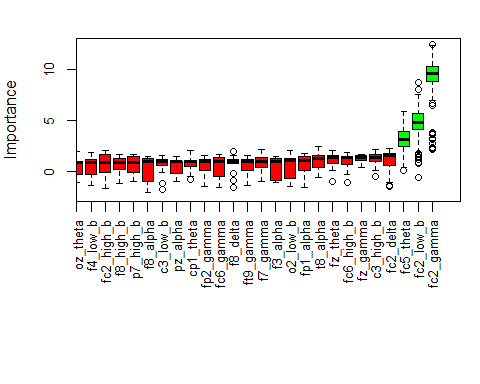
{=openxml} <w:p> <w:pPr> <w:spacing w:before="0" w:after="60"/> <w:keepNext/> <w:jc w:val="start"/> <w:pStyle w:val="caption"/> </w:pPr> <w:r> <w:rPr> <w:rFonts w:ascii="Calibri" w:hAnsi="Calibri"/> <w:sz w:val="24"/> </w:rPr> <w:t xml:space="preserve">Table </w:t> </w:r> <w:r> <w:fldChar w:fldCharType="begin" w:dirty="true"/> </w:r> <w:r> <w:instrText xml:space="preserve" w:dirty="true"> SEQ Table \* ARABIC </w:instrText> </w:r> <w:r> <w:fldChar w:fldCharType="separate" w:dirty="true"/> </w:r> <w:r> <w:rPr> <w:noProof/> <w:rFonts w:ascii="Calibri" w:hAnsi="Calibri"/> <w:sz w:val="24"/> </w:rPr> <w:t xml:space="default">1</w:t> </w:r> <w:r> <w:fldChar w:fldCharType="end" w:dirty="true"/> </w:r> <w:r> <w:rPr> <w:rFonts w:ascii="Calibri" w:hAnsi="Calibri"/> <w:sz w:val="24"/> </w:rPr> <w:t xml:space="preserve">: </w:t> </w:r> <w:r> <w:rPr> <w:b w:val="true"/> <w:rFonts w:ascii="Calibri" w:hAnsi="Calibri"/> <w:sz w:val="24"/> <w:color w:val="333333"/> </w:rPr> <w:t xml:space="preserve">Common important variables</w:t> </w:r> </w:p><w:p> <w:pPr> <w:spacing w:before="0" w:after="60"/> <w:keepNext/> <w:jc w:val="start"/> <w:pStyle w:val="caption"/> </w:pPr> <w:r> <w:rPr> <w:i/> <w:rFonts w:ascii="Calibri" w:hAnsi="Calibri"/> <w:sz w:val="20"/> <w:color w:val="333333"/> </w:rPr> <w:t xml:space="preserve">Boruta iteration on 5 seeds</w:t> </w:r> </w:p><w:tbl xmlns:w="http://schemas.openxmlformats.org/wordprocessingml/2006/main" xmlns:wp="http://schemas.openxmlformats.org/drawingml/2006/wordprocessingDrawing" xmlns:r="http://schemas.openxmlformats.org/officeDocument/2006/relationships" xmlns:w14="http://schemas.microsoft.com/office/word/2010/wordml"><w:tblPr><w:tblCellMar><w:top w:w="0" w:type="dxa"></w:top><w:bottom w:w="0" w:type="dxa"></w:bottom><w:start w:w="60" w:type="dxa"></w:start><w:end w:w="60" w:type="dxa"></w:end></w:tblCellMar><w:tblW w:type="auto" w:w="0"></w:tblW><w:tblLook w:firstRow="0" w:lastRow="0" w:firstColumn="0" w:lastColumn="0" w:noHBand="0" w:noVBand="0"></w:tblLook><w:jc w:val="center"></w:jc></w:tblPr><w:tr><w:trPr><w:cantSplit></w:cantSplit><w:tblHeader></w:tblHeader></w:trPr><w:tc><w:tcPr><w:tcBorders><w:top w:val="single" w:sz="16" w:space="0" w:color="D3D3D3"></w:top><w:bottom w:val="single" w:sz="16" w:space="0" w:color="D3D3D3"></w:bottom><w:start w:val="single" w:space="0" w:color="D3D3D3"></w:start></w:tcBorders></w:tcPr><w:p> <w:pPr> <w:spacing w:before="0" w:after="60"/> <w:keepNext/> <w:jc w:val="start"/> </w:pPr> <w:r> <w:rPr> <w:b w:val="true"/> <w:rFonts w:ascii="Calibri" w:hAnsi="Calibri"/> <w:sz w:val="20"/> </w:rPr> <w:t xml:space="preserve">Variable</w:t> </w:r> </w:p></w:tc><w:tc><w:tcPr><w:tcBorders><w:top w:val="single" w:sz="16" w:space="0" w:color="D3D3D3"></w:top><w:bottom w:val="single" w:sz="16" w:space="0" w:color="D3D3D3"></w:bottom></w:tcBorders></w:tcPr><w:p> <w:pPr> <w:spacing w:before="0" w:after="60"/> <w:keepNext/> <w:jc w:val="end"/> </w:pPr> <w:r> <w:rPr> <w:b w:val="true"/> <w:rFonts w:ascii="Calibri" w:hAnsi="Calibri"/> <w:sz w:val="20"/> </w:rPr> <w:t xml:space="preserve">MeanImp 1</w:t> </w:r> </w:p></w:tc><w:tc><w:tcPr><w:tcBorders><w:top w:val="single" w:sz="16" w:space="0" w:color="D3D3D3"></w:top><w:bottom w:val="single" w:sz="16" w:space="0" w:color="D3D3D3"></w:bottom></w:tcBorders></w:tcPr><w:p> <w:pPr> <w:spacing w:before="0" w:after="60"/> <w:keepNext/> <w:jc w:val="end"/> </w:pPr> <w:r> <w:rPr> <w:b w:val="true"/> <w:rFonts w:ascii="Calibri" w:hAnsi="Calibri"/> <w:sz w:val="20"/> </w:rPr> <w:t xml:space="preserve">MeanImp 2</w:t> </w:r> </w:p></w:tc><w:tc><w:tcPr><w:tcBorders><w:top w:val="single" w:sz="16" w:space="0" w:color="D3D3D3"></w:top><w:bottom w:val="single" w:sz="16" w:space="0" w:color="D3D3D3"></w:bottom></w:tcBorders></w:tcPr><w:p> <w:pPr> <w:spacing w:before="0" w:after="60"/> <w:keepNext/> <w:jc w:val="end"/> </w:pPr> <w:r> <w:rPr> <w:b w:val="true"/> <w:rFonts w:ascii="Calibri" w:hAnsi="Calibri"/> <w:sz w:val="20"/> </w:rPr> <w:t xml:space="preserve">MeanImp 3</w:t> </w:r> </w:p></w:tc><w:tc><w:tcPr><w:tcBorders><w:top w:val="single" w:sz="16" w:space="0" w:color="D3D3D3"></w:top><w:bottom w:val="single" w:sz="16" w:space="0" w:color="D3D3D3"></w:bottom></w:tcBorders></w:tcPr><w:p> <w:pPr> <w:spacing w:before="0" w:after="60"/> <w:keepNext/> <w:jc w:val="end"/> </w:pPr> <w:r> <w:rPr> <w:b w:val="true"/> <w:rFonts w:ascii="Calibri" w:hAnsi="Calibri"/> <w:sz w:val="20"/> </w:rPr> <w:t xml:space="preserve">MeanImp 4</w:t> </w:r> </w:p></w:tc><w:tc><w:tcPr><w:tcBorders><w:top w:val="single" w:sz="16" w:space="0" w:color="D3D3D3"></w:top><w:bottom w:val="single" w:sz="16" w:space="0" w:color="D3D3D3"></w:bottom><w:end w:val="single" w:space="0" w:color="D3D3D3"></w:end></w:tcBorders></w:tcPr><w:p> <w:pPr> <w:spacing w:before="0" w:after="60"/> <w:keepNext/> <w:jc w:val="end"/> </w:pPr> <w:r> <w:rPr> <w:b w:val="true"/> <w:rFonts w:ascii="Calibri" w:hAnsi="Calibri"/> <w:sz w:val="20"/> </w:rPr> <w:t xml:space="preserve">MeanImp 5</w:t> </w:r> </w:p></w:tc></w:tr><w:tr><w:trPr><w:cantSplit></w:cantSplit></w:trPr><w:tc><w:tcPr><w:tcBorders><w:top w:val="single" w:space="0" w:color="D3D3D3"></w:top><w:bottom w:val="single" w:space="0" w:color="D3D3D3"></w:bottom><w:start w:val="single" w:space="0" w:color="D3D3D3"></w:start><w:end w:val="single" w:space="0" w:color="D3D3D3"></w:end></w:tcBorders></w:tcPr><w:p> <w:pPr> <w:spacing w:before="0" w:after="60"/> <w:keepNext/> <w:jc w:val="start"/> </w:pPr> <w:r> <w:rPr> <w:rFonts w:ascii="Calibri" w:hAnsi="Calibri"/> <w:sz w:val="20"/> </w:rPr> <w:t xml:space="default">fc2_gamma</w:t> </w:r> </w:p></w:tc><w:tc><w:tcPr><w:tcBorders><w:top w:val="single" w:space="0" w:color="D3D3D3"></w:top><w:bottom w:val="single" w:space="0" w:color="D3D3D3"></w:bottom><w:start w:val="single" w:space="0" w:color="D3D3D3"></w:start><w:end w:val="single" w:space="0" w:color="D3D3D3"></w:end></w:tcBorders></w:tcPr><w:p> <w:pPr> <w:spacing w:before="0" w:after="60"/> <w:keepNext/> <w:jc w:val="end"/> </w:pPr> <w:r> <w:rPr> <w:rFonts w:ascii="Calibri" w:hAnsi="Calibri"/> <w:sz w:val="20"/> </w:rPr> <w:t xml:space="default">9.236019</w:t> </w:r> </w:p></w:tc><w:tc><w:tcPr><w:tcBorders><w:top w:val="single" w:space="0" w:color="D3D3D3"></w:top><w:bottom w:val="single" w:space="0" w:color="D3D3D3"></w:bottom><w:start w:val="single" w:space="0" w:color="D3D3D3"></w:start><w:end w:val="single" w:space="0" w:color="D3D3D3"></w:end></w:tcBorders></w:tcPr><w:p> <w:pPr> <w:spacing w:before="0" w:after="60"/> <w:keepNext/> <w:jc w:val="end"/> </w:pPr> <w:r> <w:rPr> <w:rFonts w:ascii="Calibri" w:hAnsi="Calibri"/> <w:sz w:val="20"/> </w:rPr> <w:t xml:space="default">8.051649</w:t> </w:r> </w:p></w:tc><w:tc><w:tcPr><w:tcBorders><w:top w:val="single" w:space="0" w:color="D3D3D3"></w:top><w:bottom w:val="single" w:space="0" w:color="D3D3D3"></w:bottom><w:start w:val="single" w:space="0" w:color="D3D3D3"></w:start><w:end w:val="single" w:space="0" w:color="D3D3D3"></w:end></w:tcBorders></w:tcPr><w:p> <w:pPr> <w:spacing w:before="0" w:after="60"/> <w:keepNext/> <w:jc w:val="end"/> </w:pPr> <w:r> <w:rPr> <w:rFonts w:ascii="Calibri" w:hAnsi="Calibri"/> <w:sz w:val="20"/> </w:rPr> <w:t xml:space="default">8.265707</w:t> </w:r> </w:p></w:tc><w:tc><w:tcPr><w:tcBorders><w:top w:val="single" w:space="0" w:color="D3D3D3"></w:top><w:bottom w:val="single" w:space="0" w:color="D3D3D3"></w:bottom><w:start w:val="single" w:space="0" w:color="D3D3D3"></w:start><w:end w:val="single" w:space="0" w:color="D3D3D3"></w:end></w:tcBorders></w:tcPr><w:p> <w:pPr> <w:spacing w:before="0" w:after="60"/> <w:keepNext/> <w:jc w:val="end"/> </w:pPr> <w:r> <w:rPr> <w:rFonts w:ascii="Calibri" w:hAnsi="Calibri"/> <w:sz w:val="20"/> </w:rPr> <w:t xml:space="default">7.26614</w:t> </w:r> </w:p></w:tc><w:tc><w:tcPr><w:tcBorders><w:top w:val="single" w:space="0" w:color="D3D3D3"></w:top><w:bottom w:val="single" w:space="0" w:color="D3D3D3"></w:bottom><w:start w:val="single" w:space="0" w:color="D3D3D3"></w:start><w:end w:val="single" w:space="0" w:color="D3D3D3"></w:end></w:tcBorders></w:tcPr><w:p> <w:pPr> <w:spacing w:before="0" w:after="60"/> <w:keepNext/> <w:jc w:val="end"/> </w:pPr> <w:r> <w:rPr> <w:rFonts w:ascii="Calibri" w:hAnsi="Calibri"/> <w:sz w:val="20"/> </w:rPr> <w:t xml:space="default">9.466658</w:t> </w:r> </w:p></w:tc></w:tr></w:tbl>

## VLF

## Boruta performed 48 iterations in 0.595289 secs.
## No attributes deemed important.
## 204 attributes confirmed unimportant: c3_alpha, c3_delta, c3_gamma,
## c3_high_b, c3_low_b and 199 more;
## Boruta performed 48 iterations in 0.595289 secs.
## No attributes deemed important.
## 204 attributes confirmed unimportant: c3_alpha, c3_delta, c3_gamma,
## c3_high_b, c3_low_b and 199 more;


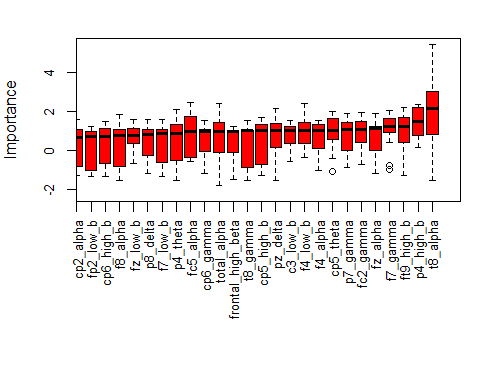


## Boruta performed 15 iterations in 0.2961578 secs.
## No attributes deemed important.
## 204 attributes confirmed unimportant: c3_alpha, c3_delta, c3_gamma,
## c3_high_b, c3_low_b and 199 more;
## Boruta performed 15 iterations in 0.2961578 secs.
## No attributes deemed important.
## 204 attributes confirmed unimportant: c3_alpha, c3_delta, c3_gamma,
## c3_high_b, c3_low_b and 199 more;


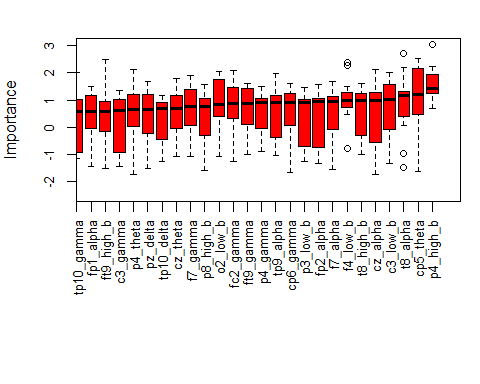


## Boruta performed 15 iterations in 0.2982259 secs.
## No attributes deemed important.
## 204 attributes confirmed unimportant: c3_alpha, c3_delta, c3_gamma,
## c3_high_b, c3_low_b and 199 more;
## Boruta performed 15 iterations in 0.2982259 secs.
## No attributes deemed important.
## 204 attributes confirmed unimportant: c3_alpha, c3_delta, c3_gamma,
## c3_high_b, c3_low_b and 199 more;


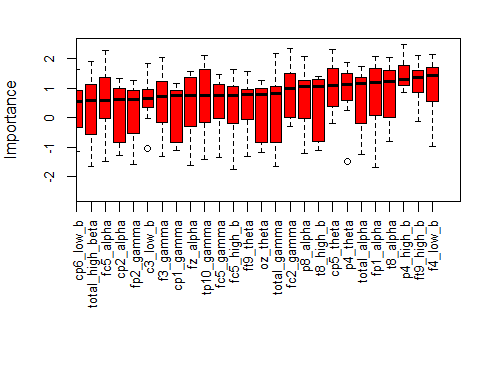


## Boruta performed 999 iterations in 7.527266 secs.
## 2 attributes confirmed important: cp5_theta, p4_high_b;
## 200 attributes confirmed unimportant: c3_alpha, c3_delta, c3_gamma,
## c3_high_b, c3_low_b and 195 more;
## 2 tentative attributes left: f7_gamma, p4_theta;
## Boruta performed 999 iterations in 7.527266 secs.
## Tentatives roughfixed over the last 999 iterations.
## 3 attributes confirmed important: cp5_theta, f7_gamma, p4_high_b;
## 201 attributes confirmed unimportant: c3_alpha, c3_delta, c3_gamma,
## c3_high_b, c3_low_b and 196 more;


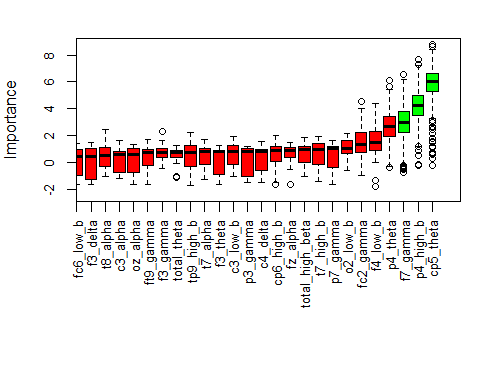


## Boruta performed 39 iterations in 0.463244 secs.
## No attributes deemed important.
## 204 attributes confirmed unimportant: c3_alpha, c3_delta, c3_gamma,
## c3_high_b, c3_low_b and 199 more;
## Boruta performed 39 iterations in 0.463244 secs.
## No attributes deemed important.
## 204 attributes confirmed unimportant: c3_alpha, c3_delta, c3_gamma,
## c3_high_b, c3_low_b and 199 more;


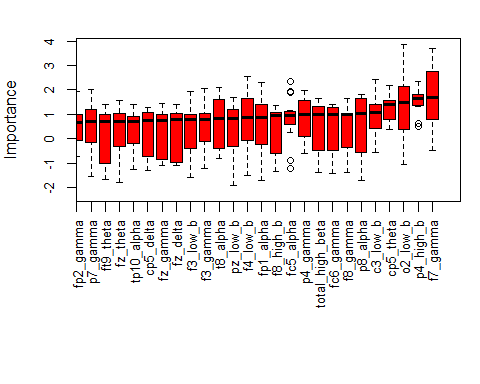
{=openxml} <w:p> <w:pPr> <w:spacing w:before="0" w:after="60"/> <w:keepNext/> <w:jc w:val="start"/> <w:pStyle w:val="caption"/> </w:pPr> <w:r> <w:rPr> <w:rFonts w:ascii="Calibri" w:hAnsi="Calibri"/> <w:sz w:val="24"/> </w:rPr> <w:t xml:space="preserve">Table </w:t> </w:r> <w:r> <w:fldChar w:fldCharType="begin" w:dirty="true"/> </w:r> <w:r> <w:instrText xml:space="preserve" w:dirty="true"> SEQ Table \* ARABIC </w:instrText> </w:r> <w:r> <w:fldChar w:fldCharType="separate" w:dirty="true"/> </w:r> <w:r> <w:rPr> <w:noProof/> <w:rFonts w:ascii="Calibri" w:hAnsi="Calibri"/> <w:sz w:val="24"/> </w:rPr> <w:t xml:space="default">1</w:t> </w:r> <w:r> <w:fldChar w:fldCharType="end" w:dirty="true"/> </w:r> <w:r> <w:rPr> <w:rFonts w:ascii="Calibri" w:hAnsi="Calibri"/> <w:sz w:val="24"/> </w:rPr> <w:t xml:space="preserve">: </w:t> </w:r> <w:r> <w:rPr> <w:b w:val="true"/> <w:rFonts w:ascii="Calibri" w:hAnsi="Calibri"/> <w:sz w:val="24"/> <w:color w:val="333333"/> </w:rPr> <w:t xml:space="preserve">Common important variables</w:t> </w:r> </w:p><w:p> <w:pPr> <w:spacing w:before="0" w:after="60"/> <w:keepNext/> <w:jc w:val="start"/> <w:pStyle w:val="caption"/> </w:pPr> <w:r> <w:rPr> <w:i/> <w:rFonts w:ascii="Calibri" w:hAnsi="Calibri"/> <w:sz w:val="20"/> <w:color w:val="333333"/> </w:rPr> <w:t xml:space="preserve">Boruta iteration on 5 seeds</w:t> </w:r> </w:p><w:tbl xmlns:w="http://schemas.openxmlformats.org/wordprocessingml/2006/main" xmlns:wp="http://schemas.openxmlformats.org/drawingml/2006/wordprocessingDrawing" xmlns:r="http://schemas.openxmlformats.org/officeDocument/2006/relationships" xmlns:w14="http://schemas.microsoft.com/office/word/2010/wordml"><w:tblPr><w:tblCellMar><w:top w:w="0" w:type="dxa"></w:top><w:bottom w:w="0" w:type="dxa"></w:bottom><w:start w:w="60" w:type="dxa"></w:start><w:end w:w="60" w:type="dxa"></w:end></w:tblCellMar><w:tblW w:type="auto" w:w="0"></w:tblW><w:tblLook w:firstRow="0" w:lastRow="0" w:firstColumn="0" w:lastColumn="0" w:noHBand="0" w:noVBand="0"></w:tblLook><w:jc w:val="center"></w:jc></w:tblPr><w:tr><w:trPr><w:cantSplit></w:cantSplit><w:tblHeader></w:tblHeader></w:trPr><w:tc><w:tcPr><w:tcBorders><w:top w:val="single" w:sz="16" w:space="0" w:color="D3D3D3"></w:top><w:bottom w:val="single" w:sz="16" w:space="0" w:color="D3D3D3"></w:bottom><w:start w:val="single" w:space="0" w:color="D3D3D3"></w:start></w:tcBorders></w:tcPr><w:p> <w:pPr> <w:spacing w:before="0" w:after="60"/> <w:keepNext/> <w:jc w:val="start"/> </w:pPr> <w:r> <w:rPr> <w:b w:val="true"/> <w:rFonts w:ascii="Calibri" w:hAnsi="Calibri"/> <w:sz w:val="20"/> </w:rPr> <w:t xml:space="preserve">Variable</w:t> </w:r> </w:p></w:tc><w:tc><w:tcPr><w:tcBorders><w:top w:val="single" w:sz="16" w:space="0" w:color="D3D3D3"></w:top><w:bottom w:val="single" w:sz="16" w:space="0" w:color="D3D3D3"></w:bottom></w:tcBorders></w:tcPr><w:p> <w:pPr> <w:spacing w:before="0" w:after="60"/> <w:keepNext/> <w:jc w:val="end"/> </w:pPr> <w:r> <w:rPr> <w:b w:val="true"/> <w:rFonts w:ascii="Calibri" w:hAnsi="Calibri"/> <w:sz w:val="20"/> </w:rPr> <w:t xml:space="preserve">MeanImp 1</w:t> </w:r> </w:p></w:tc><w:tc><w:tcPr><w:tcBorders><w:top w:val="single" w:sz="16" w:space="0" w:color="D3D3D3"></w:top><w:bottom w:val="single" w:sz="16" w:space="0" w:color="D3D3D3"></w:bottom></w:tcBorders></w:tcPr><w:p> <w:pPr> <w:spacing w:before="0" w:after="60"/> <w:keepNext/> <w:jc w:val="end"/> </w:pPr> <w:r> <w:rPr> <w:b w:val="true"/> <w:rFonts w:ascii="Calibri" w:hAnsi="Calibri"/> <w:sz w:val="20"/> </w:rPr> <w:t xml:space="preserve">MeanImp 2</w:t> </w:r> </w:p></w:tc><w:tc><w:tcPr><w:tcBorders><w:top w:val="single" w:sz="16" w:space="0" w:color="D3D3D3"></w:top><w:bottom w:val="single" w:sz="16" w:space="0" w:color="D3D3D3"></w:bottom></w:tcBorders></w:tcPr><w:p> <w:pPr> <w:spacing w:before="0" w:after="60"/> <w:keepNext/> <w:jc w:val="end"/> </w:pPr> <w:r> <w:rPr> <w:b w:val="true"/> <w:rFonts w:ascii="Calibri" w:hAnsi="Calibri"/> <w:sz w:val="20"/> </w:rPr> <w:t xml:space="preserve">MeanImp 3</w:t> </w:r> </w:p></w:tc><w:tc><w:tcPr><w:tcBorders><w:top w:val="single" w:sz="16" w:space="0" w:color="D3D3D3"></w:top><w:bottom w:val="single" w:sz="16" w:space="0" w:color="D3D3D3"></w:bottom></w:tcBorders></w:tcPr><w:p> <w:pPr> <w:spacing w:before="0" w:after="60"/> <w:keepNext/> <w:jc w:val="end"/> </w:pPr> <w:r> <w:rPr> <w:b w:val="true"/> <w:rFonts w:ascii="Calibri" w:hAnsi="Calibri"/> <w:sz w:val="20"/> </w:rPr> <w:t xml:space="preserve">MeanImp 4</w:t> </w:r> </w:p></w:tc><w:tc><w:tcPr><w:tcBorders><w:top w:val="single" w:sz="16" w:space="0" w:color="D3D3D3"></w:top><w:bottom w:val="single" w:sz="16" w:space="0" w:color="D3D3D3"></w:bottom><w:end w:val="single" w:space="0" w:color="D3D3D3"></w:end></w:tcBorders></w:tcPr><w:p> <w:pPr> <w:spacing w:before="0" w:after="60"/> <w:keepNext/> <w:jc w:val="end"/> </w:pPr> <w:r> <w:rPr> <w:b w:val="true"/> <w:rFonts w:ascii="Calibri" w:hAnsi="Calibri"/> <w:sz w:val="20"/> </w:rPr> <w:t xml:space="preserve">MeanImp 5</w:t> </w:r> </w:p></w:tc></w:tr></w:tbl>

## LF

## Boruta performed 100 iterations in 1.023577 secs.
## 5 attributes confirmed important: cp1_theta, f7_gamma, fc2_gamma,
## fz_theta, t8_alpha;
## 199 attributes confirmed unimportant: c3_alpha, c3_delta, c3_gamma,
## c3_high_b, c3_low_b and 194 more;
## Boruta performed 100 iterations in 1.023577 secs.
## 5 attributes confirmed important: cp1_theta, f7_gamma, fc2_gamma,
## fz_theta, t8_alpha;
## 199 attributes confirmed unimportant: c3_alpha, c3_delta, c3_gamma,
## c3_high_b, c3_low_b and 194 more;


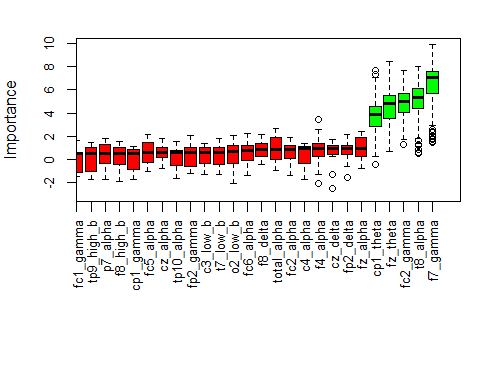


## Boruta performed 999 iterations in 7.124763 secs.
## c
## 199 attributes confirmed unimportant: c3_alpha, c3_delta, c3_gamma,
## c3_high_b, c3_low_b and 194 more;
## 1 tentative attributes left: fz_alpha;
## Boruta performed 999 iterations in 7.124763 secs.
## Tentatives roughfixed over the last 999 iterations.
## 4 attributes confirmed important: f7_gamma, fc2_gamma, fz_theta,
## t8_alpha;
## 200 attributes confirmed unimportant: c3_alpha, c3_delta, c3_gamma,
## c3_high_b, c3_low_b and 195 more;


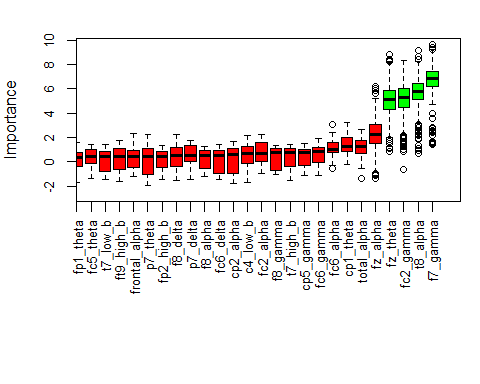


## Boruta performed 132 iterations in 1.100371 secs.
## 5 attributes confirmed important: cp1_theta, f7_gamma, fc2_gamma,
## fz_theta, t8_alpha;
## 199 attributes confirmed unimportant: c3_alpha, c3_delta, c3_gamma,
## c3_high_b, c3_low_b and 194 more;
## Boruta performed 132 iterations in 1.100371 secs.
## 5 attributes confirmed important: cp1_theta, f7_gamma, fc2_gamma,
## fz_theta, t8_alpha;
## 199 attributes confirmed unimportant: c3_alpha, c3_delta, c3_gamma,
## c3_high_b, c3_low_b and 194 more;


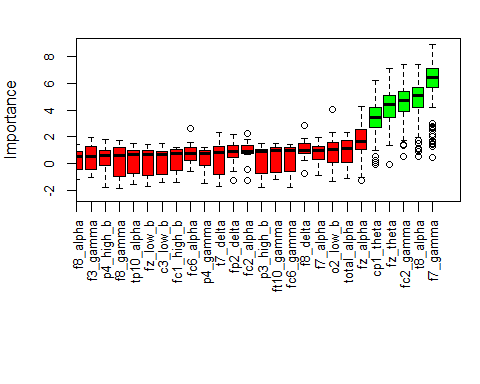


## Boruta performed 999 iterations in 7.327177 secs.
## 6 attributes confirmed important: cp1_theta, f7_gamma, fc2_gamma,
## fz_theta, t8_alpha and 1 more;
## 197 attributes confirmed unimportant: c3_alpha, c3_delta, c3_gamma,
## c3_high_b, c3_low_b and 192 more;
## 1 tentative attributes left: f8_delta;
## Boruta performed 999 iterations in 7.327177 secs.
## Tentatives roughfixed over the last 999 iterations.
## 7 attributes confirmed important: cp1_theta, f7_gamma, f8_delta,
## fc2_gamma, fz_theta and 2 more;
## 197 attributes confirmed unimportant: c3_alpha, c3_delta, c3_gamma,
## c3_high_b, c3_low_b and 192 more;


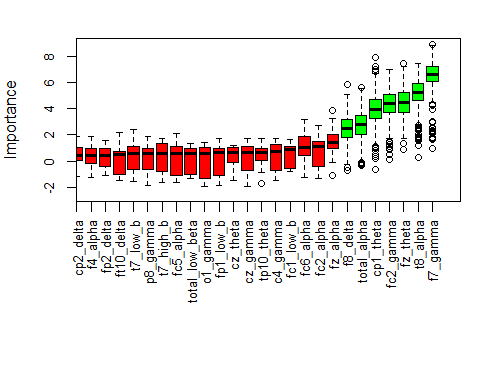


## Boruta performed 999 iterations in 7.335632 secs.
## 5 attributes confirmed important: cp1_theta, f7_gamma, fc2_gamma,
## fz_theta, t8_alpha;
## 197 attributes confirmed unimportant: c3_alpha, c3_delta, c3_gamma,
## c3_high_b, c3_low_b and 192 more;
## 2 tentative attributes left: f8_delta, total_alpha;
## Boruta performed 999 iterations in 7.335632 secs.
## Tentatives roughfixed over the last 999 iterations.
## 7 attributes confirmed important: cp1_theta, f7_gamma, f8_delta,
## fc2_gamma, fz_theta and 2 more;
## 197 attributes confirmed unimportant: c3_alpha, c3_delta, c3_gamma,
## c3_high_b, c3_low_b and 192 more;


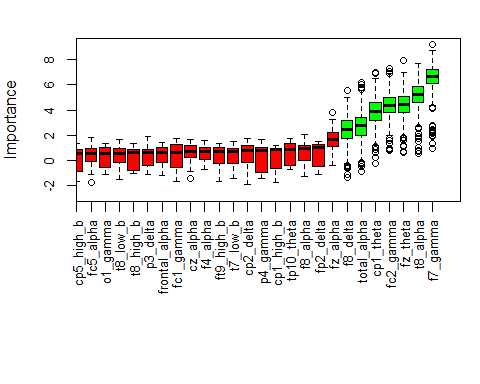
{=openxml} <w:p>

## HF

## Boruta performed 129 iterations in 1.161706 secs.
## 6 attributes confirmed important: fc2_gamma, fc2_high_b, fc2_low_b,
## fc5_theta, oz_theta and 1 more;
## 198 attributes confirmed unimportant: c3_alpha, c3_delta, c3_gamma,
## c3_high_b, c3_low_b and 193 more;
## Boruta performed 129 iterations in 1.161706 secs.
## 6 attributes confirmed important: fc2_gamma, fc2_high_b, fc2_low_b,
## fc5_theta, oz_theta and 1 more;
## 198 attributes confirmed unimportant: c3_alpha, c3_delta, c3_gamma,
## c3_high_b, c3_low_b and 193 more;


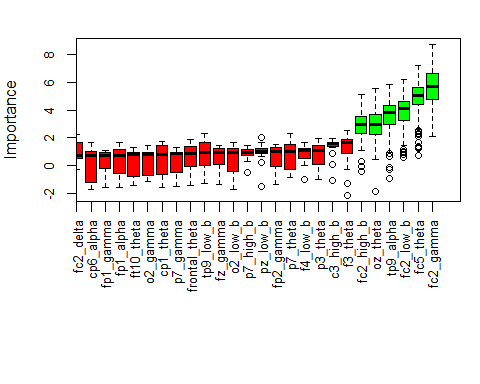


## Boruta performed 253 iterations in 2.025059 secs.
## 5 attributes confirmed important: fc2_delta, fc2_gamma, fc2_high_b,
## fc2_low_b, fc5_theta;
## 199 attributes confirmed unimportant: c3_alpha, c3_delta, c3_gamma,
## c3_high_b, c3_low_b and 194 more;
## Boruta performed 253 iterations in 2.025059 secs.
## 5 attributes confirmed important: fc2_delta, fc2_gamma, fc2_high_b,
## fc2_low_b, fc5_theta;
## 199 attributes confirmed unimportant: c3_alpha, c3_delta, c3_gamma,
## c3_high_b, c3_low_b and 194 more;


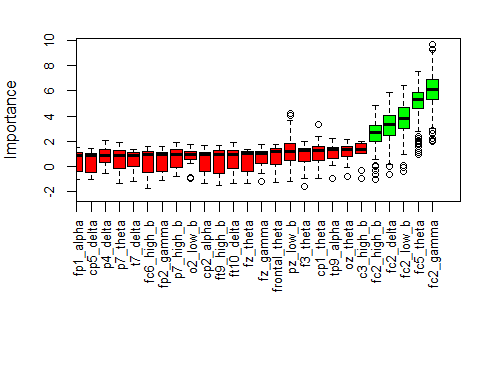


## Boruta performed 267 iterations in 2.311996 secs.
## 7 attributes confirmed important: c3_high_b, f3_theta, fc2_delta,
## fc2_gamma, fc2_low_b and 2 more;
## 197 attributes confirmed unimportant: c3_alpha, c3_delta, c3_gamma,
## c3_low_b, c3_theta and 192 more;
## Boruta performed 267 iterations in 2.311996 secs.
## 7 attributes confirmed important: c3_high_b, f3_theta, fc2_delta,
## fc2_gamma, fc2_low_b and 2 more;
## 197 attributes confirmed unimportant: c3_alpha, c3_delta, c3_gamma,
## c3_low_b, c3_theta and 192 more;


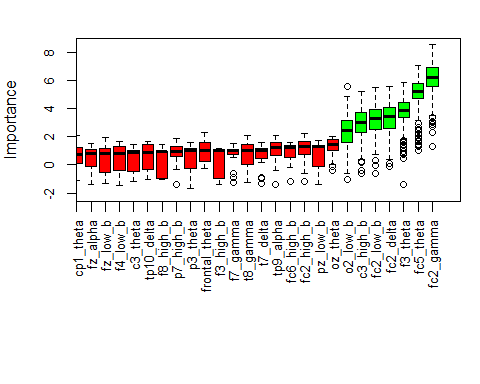


## Boruta performed 97 iterations in 0.9257271 secs.
## 5 attributes confirmed important: c3_high_b, fc2_delta, fc2_gamma,
## fc2_low_b, fc5_theta;
## 199 attributes confirmed unimportant: c3_alpha, c3_delta, c3_gamma,
## c3_low_b, c3_theta and 194 more;
## Boruta performed 97 iterations in 0.9257271 secs.
## 5 attributes confirmed important: c3_high_b, fc2_delta, fc2_gamma,
## fc2_low_b, fc5_theta;
## 199 attributes confirmed unimportant: c3_alpha, c3_delta, c3_gamma,
## c3_low_b, c3_theta and 194 more;


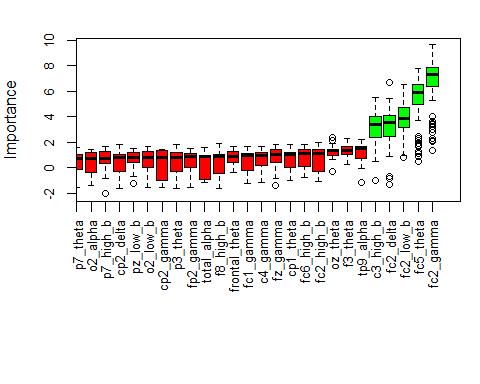


## Boruta performed 280 iterations in 2.231043 secs.
## 3 attributes confirmed important: fc2_gamma, fc2_high_b, fc5_theta;
## 201 attributes confirmed unimportant: c3_alpha, c3_delta, c3_gamma,
## c3_high_b, c3_low_b and 196 more;
## Boruta performed 280 iterations in 2.231043 secs.
## 3 attributes confirmed important: fc2_gamma, fc2_high_b, fc5_theta;
## 201 attributes confirmed unimportant: c3_alpha, c3_delta, c3_gamma,
## c3_high_b, c3_low_b and 196 more;


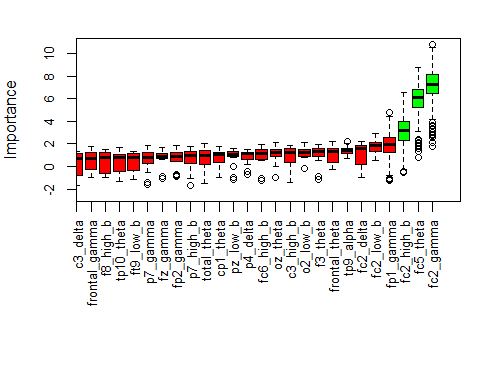
{=openxml} <w:p> <w:pPr> <w:spacing w:before="0" w:after="60"/> <w:

## Boruta performed 999 iterations in 7.881231 secs.
## 4 attributes confirmed important: cp6_high_b, f7_gamma, fc2_delta,
## fz_theta;
## 199 attributes confirmed unimportant: c3_alpha, c3_delta, c3_gamma,
## c3_high_b, c3_low_b and 194 more;
## 1 tentative attributes left: p8_delta;
## Boruta performed 999 iterations in 7.881231 secs.
## Tentatives roughfixed over the last 999 iterations.
## 5 attributes confirmed important: cp6_high_b, f7_gamma, fc2_delta,
## fz_theta, p8_delta;
## 199 attributes confirmed unimportant: c3_alpha, c3_delta, c3_gamma,
## c3_high_b, c3_low_b and 194 more;


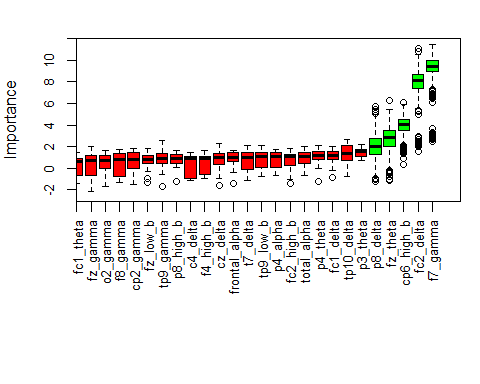


## Boruta performed 999 iterations in 7.358049 secs.
## 4 attributes confirmed important: f7_gamma, fc2_delta, fz_theta,
## tp10_delta;
## 199 attributes confirmed unimportant: c3_alpha, c3_delta, c3_gamma,
## c3_high_b, c3_low_b and 194 more;
## 1 tentative attributes left: fc2_low_b;
## Boruta performed 999 iterations in 7.358049 secs.
## Tentatives roughfixed over the last 999 iterations.
## 4 attributes confirmed important: f7_gamma, fc2_delta, fz_theta,
## tp10_delta;
## 200 attributes confirmed unimportant: c3_alpha, c3_delta, c3_gamma,
## c3_high_b, c3_low_b and 195 more;


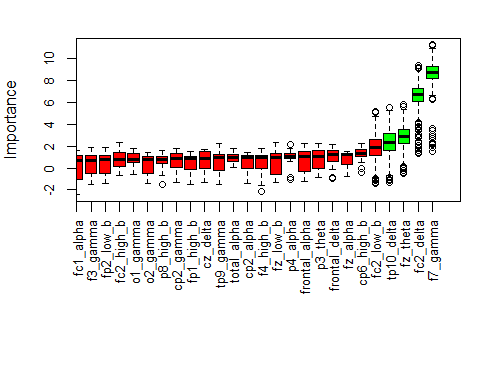


## Boruta performed 999 iterations in 7.114521 secs.
## 4 attributes confirmed important: cp6_high_b, f7_gamma, fc2_delta,
## tp10_delta;
## 199 attributes confirmed unimportant: c3_alpha, c3_delta, c3_gamma,
## c3_high_b, c3_low_b and 194 more;
## 1 tentative attributes left: fc1_delta;
## Boruta performed 999 iterations in 7.114521 secs.
## Tentatives roughfixed over the last 999 iterations.
## 5 attributes confirmed important: cp6_high_b, f7_gamma, fc1_delta,
## fc2_delta, tp10_delta;
## 199 attributes confirmed unimportant: c3_alpha, c3_delta, c3_gamma,
## c3_high_b, c3_low_b and 194 more;


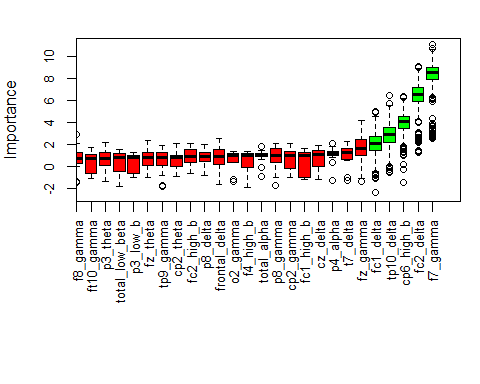


## Boruta performed 930 iterations in 6.901321 secs.
## 2 attributes confirmed important: f7_gamma, fc2_delta;
## 202 attributes confirmed unimportant: c3_alpha, c3_delta, c3_gamma,
## c3_high_b, c3_low_b and 197 more;
## Boruta performed 930 iterations in 6.901321 secs.
## 2 attributes confirmed important: f7_gamma, fc2_delta;
## 202 attributes confirmed unimportant: c3_alpha, c3_delta, c3_gamma,
## c3_high_b, c3_low_b and 197 more;


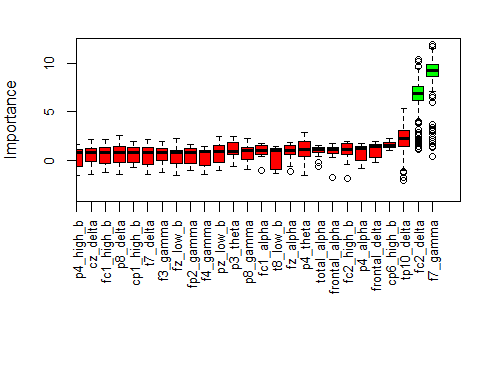


## Boruta performed 520 iterations in 3.903469 secs.
## 5 attributes confirmed important: f7_gamma, fc2_delta, fc2_high_b,
## t7_delta, tp10_delta;
## 199 attributes confirmed unimportant: c3_alpha, c3_delta, c3_gamma,
## c3_high_b, c3_low_b and 194 more;
## Boruta performed 520 iterations in 3.903469 secs.
## 5 attributes confirmed important: f7_gamma, fc2_delta, fc2_high_b,
## t7_delta, tp10_delta;
## 199 attributes confirmed unimportant: c3_alpha, c3_delta, c3_gamma,
## c3_high_b, c3_low_b and 194 more;


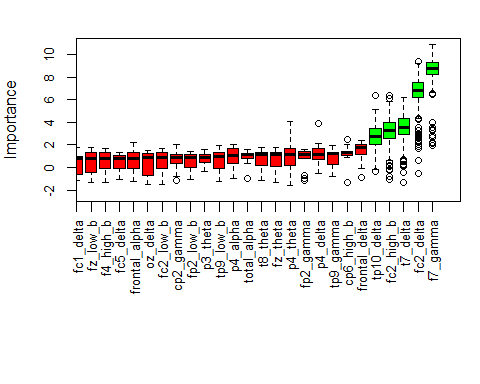
{=openxml} <w:p> <w:pPr> <w:spacing w:before="0" w:after="60"/> <w:keepNext/> <w:jc
